# A Multi-Scale and Multi-Technique Approach for the Characterization of the Effects of Spatially Fractionated X-ray Radiation Therapies in a Preclinical Model

**DOI:** 10.3390/cancers13194953

**Published:** 2021-10-01

**Authors:** Mariele Romano, Alberto Bravin, Alberto Mittone, Alicia Eckhardt, Giacomo E. Barbone, Lucie Sancey, Julien Dinkel, Stefan Bartzsch, Jens Ricke, Marianna Alunni-Fabbroni, Heidrun Hirner-Eppeneder, Dmitry Karpov, Cinzia Giannini, Oliver Bunk, Audrey Bouchet, Viktoria Ruf, Armin Giese, Paola Coan

**Affiliations:** 1 Department of Medical Physics, Faculty of Physics, Ludwig-Maximilians-Universität, Am Coulombwall 1, München, 85748 Garching, Germany; mariele.romano@physik.uni-muenchen.de (M.R.); a.eckhardt@uke.de (A.E.); giacomo.barbone@lmu.de (G.E.B.); 2 European Synchrotron Radiation Facility, 71 Avenue des Martyrs, 38000 Grenoble, France; bravin@esrf.fr (A.B.); amittone@cells.es (A.M.); dmitry.karpov@esrf.fr (D.K.); 3 Department of Physics, Faculty of Physics, University of Milano-Bicocca, 20126 Milan, Italy; 4 CELLS-ALBA Synchrotron, 08290 Cerdanyola del Valles, Spain; 5 Department of Radiology, University Hospital, Ludwig-Maximilians-Universität, 81377 Munich, Germany; Julien.Dinkel@med.uni-muenchen.de (J.D.); jens.ricke@med.uni-muenchen.de (J.R.); Marianna.Alunni@med.uni-muenchen.de (M.A.-F.); Heidrun.Hirner-Eppeneder@med.uni-muenchen.de (H.H.-E.); 6 Centre de Recherche UGA/INSERM U1209/CNRS UMR5309, Institute for Advanced Biosciences, 38700 La Tronche, France; Lucie.sancey@univ-grenoble-alpes.fr; 7 Department of Radiation Oncology, School of Medicine, Technical University of Munich, Klinikum Rechts der Isar, 81675 Munich, Germany; stefan.bartzsch@tum.de; 8 Department of Radiation Sciences (DRS), Institute of Radiation Medicine (IRM), Helmholtz Zentrum München, 85764 Neuherberg, Germany; 9 Swiss Light Source, Paul Scherrer Institute, 5232 Villigen, Switzerland; oliver.bunk@psi.ch; 10 Institute of Crystallography, National Research Council, 70126 Bari, Italy; cinzia.giannini@ic.cnr.it; 11 Inserm U1296 Unit “Radiation: Defense, Health Environment”, 69008 Lyon, France; audrey.bouchet@inserm.fr; 12 Center for Neuropathology and Prion Research, Ludwig-Maximilians-Universität, 81377 Munich, Germany; Viktoria.Ruf@med.uni-muenchen.de (V.R.); Armin.Giese@med.uni-muenchen.de (A.G.)

**Keywords:** X-ray phase-contrast imaging, glioblastoma, animal model, hydroxyapatite, virtual histology, FLASH, spatially fractionated radiotherapy, MRT

## Abstract

**Simple Summary:**

This study aims at using a multi-technique approach to detect and analyze the effects of high dose rate spatially fractionated radiation therapies and to compare them to seamless broad beam irradiation targeting healthy and glioblastoma-bearing rat brains and delivering three different doses per each irradiation geometry. Brains were analyzed post mortem by multi-scale X-ray phase contrast imaging–computed tomography, histology, immunohistochemistry, X-ray fluorescence, and small- and wide-angle X-ray scattering to achieve detailed visualization, characterization and classification in 3D of the radio-induced effects on brain tissues. The original results bring new insights to the understanding of the response of cerebral tissue and tumors treated with high dose rate spatially fractioned radiotherapies and put the basis for channeling studies of in-vivo applications for monitoring RT effects.

**Abstract:**

The purpose of this study is to use a multi-technique approach to detect the effects of spatially fractionated X-ray Microbeam (MRT) and Minibeam Radiation Therapy (MB) and to compare them to seamless Broad Beam (BB) irradiation. Healthy- and Glioblastoma (GBM)-bearing male Fischer rats were irradiated in-vivo on the right brain hemisphere with MRT, MB and BB delivering three different doses for each irradiation geometry. Brains were analyzed post mortem by multi-scale X-ray Phase Contrast Imaging–Computed Tomography (XPCI-CT), histology, immunohistochemistry, X-ray Fluorescence (XRF), Small- and Wide-Angle X-ray Scattering (SAXS/WAXS). XPCI-CT discriminates with high sensitivity the effects of MRT, MB and BB irradiations on both healthy and GBM-bearing brains producing a first-time 3D visualization and morphological analysis of the radio-induced lesions, MRT and MB induced tissue ablations, the presence of hyperdense deposits within specific areas of the brain and tumor evolution or regression with respect to the evaluation made few days post-irradiation with an in-vivo magnetic resonance imaging session. Histology, immunohistochemistry, SAXS/WAXS and XRF allowed identification and classification of these deposits as hydroxyapatite crystals with the coexistence of Ca, P and Fe mineralization, and the multi-technique approach enabled the realization, for the first time, of the map of the differential radiosensitivity of the different brain areas treated with MRT and MB. 3D XPCI-CT datasets enabled also the quantification of tumor volumes and Ca/Fe deposits and their full-organ visualization. The multi-scale and multi-technique approach enabled a detailed visualization and classification in 3D of the radio-induced effects on brain tissues bringing new essential information towards the clinical implementation of the MRT and MB radiation therapy techniques.

## 1. Introduction

Glioblastoma (GBM) is the most common and aggressive intra-axial primary tumor, accounting for about 60% of the cases [1] and causing around 2.7% of all cancer-related deaths [2]. It has a poor prognosis with a survival ranging from 7 months [3] to 12–15 months [4,5] with a long-term survival probability less than 3% [6]. GBM treatment involves surgery, chemotherapy, and radiation therapy (RT); due to the radio- and chemo-resistance and highly infiltrative growth of GBM, present treatments are only able to slow down the development of the disease permitting an increase of the survival by a few months [7]. For all these reasons, there is the need for an effective therapy for the management of gliomas.

In the last 25 years, a spatially fractionated RT called Microbeam Radiation Therapy (MRT) was introduced and developed as an alternative for tumor treatments [8,9]. MRT was proven to be well tolerated by healthy tissue while being highly effective on tumor control (in terms of tumor growth delay or complete tumor sterilization) [10]. MRT uses an array of quasi-parallel X-ray beamlets of width in the 25–100 µm range and inter-microbeam centre-to-centre (c-t-c) spacing between 100 and 400 µm, producing a non-homogeneous dose deposition of alternating peaks and valleys delivering peak doses up to hundreds of Gy in a unique fraction. X-ray beams produced at third and fourth generation synchrotron facilities are particularly well adapted for producing the beamlets for MRT because of their inherently high collimation and dose rates of several orders of magnitude larger than conventional sources. These properties allow the delivery of radiation locally in micrometric windows at high speed, preventing beam smearing due to the cardio-synchronous pulsations [11,12].

Simultaneously, X-ray Minibeam (MB) radiation therapy was developed as a RT with lateral dose profile similar to MRT but with larger beams and c-t-c distances in order to overcome the problem of possible radiation smearing, as well as to reduce the stringent requirements of MRT in terms of dose rates [13]. The effectiveness of MRT and MB fractioned treatments relies on the so-called dose-volume effect [14]: doses of hundreds of Gray are well tolerated if delivered in micrometric beamlets and produce a preferential effect on tumoral vasculature rather than on healthy vessel network [15,16,17]. Despite that, the overall biological response to these RTs of the irradiated tissues is still not completely known and imaging techniques reaching the sub-micron spatial resolution in three-dimensions (3D) are keys in identifying and classifying the MRT- and MB-induced effects on brain tissues.

X-ray Phase Contrast Imaging–Computed Tomography (XPCI-CT) [18,19] is a powerful imaging technique for post mortem radiation treatment evaluation and follow-ups of even entire organs. This technique achieves micron and sub-micron resolutions and is highly sensitive in visualizing brain and CNS structures. Previous studies proved that XPCI-CT is a well-suited imaging method for multiscale neuroimaging over a broad range of applications such as Alzheimer’s disease [20,21,22], experimental autoimmune encephalomyelitis [23], brain tumor detection [24,25], small animal brain visualization within the skull [26] and human brain nano-anatomy [27]. 

In this work, we report on a multi-technique analysis performed on both healthy and GBM-bearing rat brains after treatment with either MRT, MB or standard broad beam RT (BB) for the assessment and classification of the specific radio-induced effects. Though histology remains the gold-standard technique for evaluating pathological states at high spatial resolution, it is still limited to a two-dimensional (2D) analysis and complete organ inspections need serial cutting of the tissue, which is a labor- and time-consuming sample-destructive practice. XPCI-CT, being able to provide multiscale data (from whole organs down to cellular level) with isotropic micron and submicron spatial resolutions, is here applied as a virtual histology technique for post mortem investigation in 3D of full brain organs. The XPCI-CT technique provided, for the first time, a 3D visualization and quantification of the tumor volume and of Ca/Fe deposits. In the first phase of this project, here reported, we aimed at testing the potential and the sensitivity of the methodology on the different irradiation protocols and presenting the versatile and rich analysis possibilities offered by the applied 3D imaging and multi-technique approach with respect standard histology and immunohistochemistry. Finally, it permitted to assess, for the first time, the 3D map of the radiosensitivity of the different brain areas. We postulate/propose that it can be included in the follow-up protocol for development of novel radiotherapies. We correlate XPCI-CT with histology analysis in order to benchmark the findings. Furthermore, we applied the Small- and Wide-Angle X-ray Scattering (SAXS/WAXS) and X-ray Fluorescence (XRF) techniques on specific regions of interest to complement the morphological characterization of the samples provided by XPCI-CT. SAXS/WAXS provided an accurate structural and elemental information on the radio-induced effects of the used therapies and, in particular, an in-depth study of the chemical and crystalline nature of the microcalcifications produced by both the irradiation and the tumor evolution. Thus, this study provides, for the first time, a 3D visualization, quantification and characterization of the effects of BB, MRT and MB irradiations on both healthy and GBM-bearing rat brains. The objective is to distinguish and separately examine the purely radio-induced lesions within given brain regions (visible on irradiated healthy brains) and the effects on the tumor-bearing animals of the different irradiation geometries and applied doses. 

## 2. Materials and Methods

### 2.1. Animal and Sample Preparation

Sixty-five male Fischer rats were involved in the study performed in two different experimental sessions. At the age of 8 weeks, at day 0 (D0), forty of these animals were implanted with 9L GBM cell-line according to literature protocols [28,29,30]. At D10 all rats but the healthy-controls (i.e., not implanted and not radiation treated animals) were irradiated in one shot with different irradiation protocols in-vivo at the Biomedical beamline ID17 of the European Synchrotron Radiation Facility (ESRF, Grenoble, France), as described in the section “RT protocol”. Animals were then housed and monitored at the ESRF animal facility and sacrificed at the end of the experiment or at fixed time points to study the evolution of the tumor and the radio-induced effects over time. Healthy animals were sacrificed at D138, while for each GBM-bearing group, the sacrifice day was set accordingly to the animals’ monitoring protocol including animal daily cares and in-vivo Magnetic Resonance Imaging (MRI) sessions (see details in Table 1). After animals’ euthanasia, brains were dissected out and fixed in 4% paraformaldehyde in phosphate buffered saline solution for immersion. All procedures related to animal care conformed to the guidelines of the French government and were approved and conducted under the authorization number #01261.02. 

**Figure 1:**
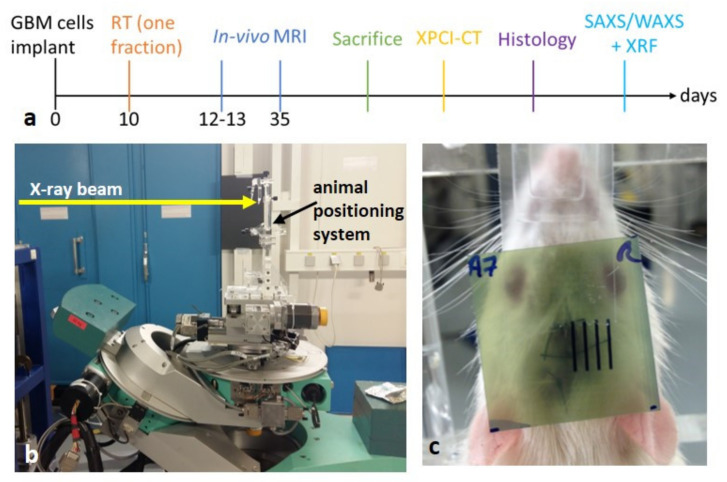
Timeline of the experimental protocol: (**a**) the days of the sacrifice depends on the groups and/or on the decided time points. Animals’ irradiation setup: the synchrotron X-ray beam, indicated by the yellow arrow, is filtered by some metal absorbers and shaped by slits collimators to the desired vertical and horizontal dimensions and the goniometer allows for the precise positioning of the animal by means of translation and rotation motor stages that are remotely controlled (**b**); picture of an irradiated rat with a GafChromic film placed just before the animal for checking the correct delivery of the X-ray beam (**c**).

Few days before the XPCI-CT imaging experiment, all the samples were dehydrated in an increasing ethanol series (50%, 60% for 4 hours) and kept in 86% ethanol. Once all samples were imaged with XPCI-CT, histology, immunohistochemistry, SAXS/WAXS and XRF analysis were performed as described in the following sections. A timeline of the experimental protocol is reported in Figure 1a.

### 2.2. RT Protocol

The irradiation sessions were performed at the MRT dedicated hutch of ID17, with the animal stage placed on a remotely controlled goniometer located at about 38.5 m from the radiation source (further details in [31]). Animals were irradiated under anesthesia with isoflurane inhalation (4% for 2 minutes) for induction followed by an intraperitoneal xylazine/ketamine injection (64.5/5.4 mg/kg). Irradiations, either BB or spatially fractionated RTs (MRT or MB), were delivered in an antero-posterior geometry placing the animals on a stereotactic frame [29], see Figure 1b. The 40 GBM-bearing animals were divided into 9 groups according to Table 1. The field of irradiation (FOI) was set to 5 × 8 mm^2^ (H × V) for BBs and MRTs and to 6.5 × 8 mm^2^ (H × V) for MBs to ensure an integer number of beamlets in the FOI, respectively 24 and 7. The same irradiation protocols were applied to the 25 healthy (no tumor implanted) rats to investigate the effects of the treatment on the healthy brain tissue. The two control groups are named healthy- and GBM-control, respectively, to avoid ambiguity.

For the MRT and MB cases, the beam was fractionated by means of a custom-made multi-slit collimator [32]. 

The beam centering procedure was achieved on each rat after having acquired a radiograph of the animal on the stereotactic frame, which allows targeting the RT beams in the same anatomical area after identification of the bregma and the irradiation coordinates following a rat atlas [33] and sparing the eye. Details on the imaging procedure for the RT target alignment are given in [34]. The irradiation area was vertically centered with the bregma, while it was displaced of 3.5 mm horizontally in order to only irradiate the right hemisphere. A GafChromic film was placed on the stereotactic frame at the beam entrance face to verify the effective irradiation (Figure 1c).

### 2.3. In-Vivo MRI Monitoring

To follow up the tumor growth and evolution, in-vivo MRI was performed on the implanted animals. Two in-vivo MRI sessions were performed at D12-13 and D35 to investigate the initial size and shape of the tumor and possible intra-animal variabilities and to check the tumor evolution. 

All MRI sessions were performed at the 4.7 T IRMaGe MRI facility (Avance III console; Bruker, Ettlingen, Germany) in Grenoble using an actively decoupled cross-coil setup. Animals were anesthetized with 4% isoflurane for induction and 2% for maintenance. The tail vein was equipped with a catheter to deliver the MRI contrast agent (Gadolinium (Gd) Dotarem®, 0.4 µL/g of the animal weight). During the whole MRI session, the rat temperature was maintained at 37.0 °C, by means of pre-heated water-filled tubes, and the breath rate at about 60 breath/min by modulating the gaseous isoflurane delivery. The following imaging sequences were applied:

Anatomical imaging was performed with a T_2_-weighted (T_2_W) spin-echo sequence: voxel size = 117 × 117 × 1000 µm^3^, 19 slices, echo time = 40 ms, flip angle = 90°; number of averages = 2, repetition time = 2500 ms, 2:40 min of total acquisition time;

T_1_-weighted (T_1_w) spin-echo sequence was performed before and 30s after the injection of the Gd contrast agent through the tail vein and flushed with 500 µL of sterile saline solution. The used parameters are: voxel size = 234 × 234 × 1000 µm^3^, 19 slices, echo time = 5, flip angle = 90°; number of averages = 4, repetition time = 800 ms, 1:17 min of total acquisition time.

### 2.4. XPCI-CT Imaging

Ex vivo propagation-based XPCI-CT was performed at ID17 (ESRF), at the TOMCAT-X02DA beamline [35,36] of the Swiss Light Source (SLS, Villigen, Switzerland) and at the P05 beamline [37,38] of the PETRA III synchrotron (Hamburg, Germany) to achieve different spatial resolutions and thus allowing a multi-scale morphological analysis. For all the imaging sessions, samples (within sealed plastic containers in an 86% ethanol bath) were placed on sophisticated motorized translation and rotation stages allowing the sample alignment with respect to the beam and detector and the CT imaging acquisitions. All measurements were performed by using monochromatic X-ray beams. Samples were first imaged at the ESRF with a 3.25 × 3.25 × 3.25 µm^3^ voxel size (in the following indicated as 3.25^3^ µm^3^, being the voxel isotropic) and afterwards, a selection of interesting cases were analyzed at the other facilities at higher spatial resolutions.

#### 2.4.1. 3.25^3^ µm^3^ Voxel Size XPCI-CT at ID17, ESRF

These scans were realized in the imaging hutch of the ID17 beamline, placed around 150 m downstream the X-ray source. The sample was illuminated by a laminar, monochromatic 35 keV X-ray beam and placed 1.8 m from the imaging detector, which is a sCMOS PCO.Edge 5.5 [39], equipped with a 50 µm-thick LuAG:Ce scintillator screen and a 2× magnification indirect conversion optics [31]. For each CT scan, 4000 projections were acquired over 360° with an exposure time per projection of 30 ms. Every rat brain was entirely imaged requiring 5–6 vertical CT scans. The CT images were reconstructed using the filtered back-projection reconstruction method and the Paganin’s phase retrieval algorithm [40], both implemented in the PyHST2 package [41].

#### 2.4.2. 1.23 µ.m^3^ Voxel Size XPCI-CT at P05, PETRA III

These scans were obtained in the micro-tomography experimental hutch (EH2) with a 30 keV X-ray beam impinging on the sample placed at 50 cm from the CMOS KIT detector. For each CT scan, 6000 projections over 720° were acquired with an exposure time of 55 ms per projection. A 5× magnification optic system was used together with a 100 μm thick LuAG:Ce single crystal scintillator. The image reconstruction was performed with dedicated MATLAB [42,43] scripts based on filtered back-projection and Paganin’s algorithm. In some cases, maximum intensity projections (MIP) of a stack of N subsequent slices were computed. The MIP creates an output image containing in each pixel the maximum value over all images in the stack at that particular pixel location.

#### 2.4.3. 0.7^3^ µm^3^ Voxel Size XPCI-CT at TOMCAT, PSI

These scans were acquired using a 21 keV monochromatic beam with a sample-to-detector distance of 5 cm. A total of 1801 angular projections were collected over 180° with an exposure time of 150 ms per projection with a PCO.Edge 5.5 camera coupled to a 20 µm thick LuAG:Ce scintillator screen and an UPLAPO10× microscope. The reconstruction procedure was performed using a specified gridrec based software [44,45] and the Paganin’s algorithm for phase retrieval.

### 2.5. Segmentation Procedure

Specific segmentation procedures to separate and quantify the volumes of the features of interest (either tumor or microcalcifications) were implemented and applied to all the 3.25^3^ µm^3^ voxel size datasets, which encompass the entire brain volume.

#### 2.5.1. Tumor Segmentation on MRI Images

The segmentation of tumor volumes was performed on T_2_w images acquired post Gd injection. Thus, the contrast agent uptake made the tumor well distinguishable from the rest of the brain tissue allowing an upper threshold-based segmentation procedure [46]. Hemorrhage was excluded by the segmentation procedure since it produces a recognizable signal, i.e., a T_2_w signal loss [47]. For every imaged sample, a threshold in the grey scale values was set to segment the Gd-loaded tumor out from the surrounding tissues in MRI images. This was realized with the Analyze Particles plugin of the Fiji software [48] returning the number of pixels of the segmented area (tumor area) on each slice. The total volume of the tumor was obtained by multiplying the tumor area by the MRI slice-image thickness. 

#### 2.5.2. Tumor Segmentation on XPCI-CT Images 

After XPCI-CT image reconstruction, each sample dataset was prepared for tumor segmentation to be performed with the ilastik software [49], as shown in Figure S1. The procedure is explained in the “tumor segmentation” section of the Supplementary Materials. The validation of the tumor segmentation is reported in Figure S2, where the segmented volume is validated by monoclonal mouse anti-glial fibrillary acidic protein (GFAP) staining on four different samples: results of this ilastik-based segmentation procedure show a good match for the GBM detection with the GFAP histology images. Nevertheless, areas with infiltrative tumor cells are overestimated by the segmentation procedure, as visible in Figure S2d–d”. The estimated tumor volumes are plotted against the animal survival; data of animals belonging to the same group, which died within 3 days, are averaged and reported as a single point.

#### 2.5.3. Compatibility Study between XPCI- and MRI-Based Tumor Volumes 

In order to include in the same analysis the tumor volumes obtained from MRI (in-vivo) and XPCI-CT (ex-vivo) images, the assessed tumor volumes were normalized by the total brain volume. This volume scaling is necessary to account for modifications (usually shrinkage) of the organ due to the tissue fixation procedures, which is a necessary step for ex-vivo experiments to avoid degradation of biological material. Before doing so, a compatibility study between the tumor volumes obtained with MRI and XPCI-CT was performed. The MB350 group was selected for this purpose since all animals in this group died by D15-16, i.e., 3–4 days after an MRI session. Among all the irradiated animal groups, the MB350 is the only one where MRI and XPCI-CT images were acquired a few days apart enabling a good comparison of the tumor volume estimations obtained with the two imaging techniques independently. To compare the assessments of the tumor volumes calculated by XPCI-CT and MRI, and thus to verify the compatibility between the two estimations, the following parameters were computed: (i) the tumor volumes, normalized to the full-brain volume, for all the seven tumor-bearing animals of the MB350 group computed both by segmenting the MRI and XPCI-CT images, together with the associated error; (ii) the compatibility reporting the *t* value, i.e., the difference of the tumor volumes obtained with the two methods divided by the error of the difference; (iii) the probability *P*(*t*) associated with the different values of *t* according to the Gaussian table; (iv) the complementary value of *P*(*t*), named confidence level (C.L.). Measures with C.L. >5% are considered to be compatible to each other.

#### 2.5.4. Segmentation of Hyperdense Structures (i.e., Microcalcifications)

For every brain in which hyperdense structures (proved to be microcalcifications) were detected, an automated threshold method was applied to segment these features from the embedding tissues. The segmentation procedure was performed with the Fiji 3D Object Counter plugin. A threshold in the grey scale was chosen for each sample to select all the microcalcifications, which are characterized by grey values in the range of (4.8–65.5) × 10^3^ for 16-bit images, and segment out their volume-data. The 3D Object counter plugin detects slice by slice the pixels above the set threshold and, afterwards, creates a volume for every single microcalcification by putting together the areas that in the different slices were assigned to the same object. At the end of the process, the plugin returns the total number of voxels composing each microcalcification. To compare the results obtained for the BB, MRT and MB groups, the total volume of the detected microcalcifications was normalized to the FOI. The volumes of microcalcifications of rats that died within three days and that belong to the same RT group, are averaged together.

#### 2.5.5. 3D Image Rendering and Computing Aspects

3D rendering of the tumor and microcalcifications image volume were realized with the software VG Studio Max 3.5 (Volume Graphics GmbH, Heidelberg, Germany) importing the full brain volume and separately the segmented tumor or microcalcification volumes.

The image processing and segmentation procedures were carried out with a Fujitsu laptop with 4 Intel Core i7 CPU processors and 2.5 GHz, a Fujitsu workstation with 8 Intel Xeon CPU processors with 4 kernels and 2.6 GHz or via the ESRF Networked Interactive Computing Environment (NICE) where larger computational power was required. The segmentation procedures of a tumor volume on MRI images, on XPCI-CTs and microcalcifications volumes can take up to about 15 min, 5 h and 90 h, respectively. Usually, when performing microcalcifications segmentation the full dataset is divided in sub volumes that are segmented separately.

### 2.6. Histology and Immunohistochemistry Analysis

All the healthy brain samples were included in paraffin blocks and 3 µm thick slices were cut with a Leica SM2010R Sliding Microtome, Leica Microsystems GmbH, Wetzlar, Deutschland to perform Hematoxylin and Eosin (H&E), Alizarin Red (for Ca deposits), Perls’ Prussian Blue (for Fe particles) and GFAP (for gliosis detection) histologic stainings. Details are given in the “Histologic procedures” section of the Supplementary Materials. 

In addition to healthy brains, the tumor bearing brains treated with MRT600 showing a small or no residual GBM on XPCI-CT images were serially cut and stained with GFAP for validating the tumor presence or sterilization. 

### 2.7. SAXS/WAXS and XRF Experiments

#### 2.7.1. Sample Preparation

For seventeen (healthy and GBM bearing) rats brain samples, 80 µm thick slices were cut with a Leica SM2010R Sliding Microtome and were prepared for the SAXS/WAXS and XRF experimental sessions placing them in sealed Ultralene sachets mounted on a custom-made and in-house designed sample holder.

#### 2.7.2. Data Acquisition

SAXS and WAXS scanning microscopy data were collected at the cSAXS beamline [50,51] using a monochromatic X-ray beam of 13.589 keV of energy and 2.4 × 10^11^ photons/s extracted by means of a liquid N_2_-cooled, fixed-exit Si(111) monochromator with bendable second crystal for horizontal focusing to about 45 µm Full Width at Half Maximum (FWHM), focused by a Rh coated mirror for vertical focusing to about 25 µm FWHM. The SAXS and WAXS maps were recorded by a Pilatus 2M area detector [52] and the XRF maps were collected with Ketek VIAMP KC00-C1T0-H030-ML8B 133 Silicon drift detector with the signal processing done by XIA FALCONX electronics. With calibration samples, energy-windows have been defined to integrate the P, Ca, and Fe signal. Samples were placed onto a motorized 2D translation stage allowing movements on the perpendicular plane with respect to the X-ray beam direction. The sample-to-detector distance was set to 7098 and 243.7 mm for SAXS in combination with XRF and WAXS, respectively, with exposure times of 0.4 s for SAXS and XRF and 0.3 s for WAXS. Data collection was performed in continuous vertical lines with the sample moving at constant speed while the detector was recording data frames with in-line rates of 1/0.405 Hz for SAXS and XRF and 1/0.305 Hz for WAXS. For SAXS data collection, a 7 m long air-evacuated flight tube was inserted between the sample and the detector. SAXS and WAXS 2D data were calibrated by silver behenate (SAXS) and NIST SRM640b (WAXS) and folded into 1D profiles. Details of the SAXS/WAXS and XRF data analysis are reported in the “SAXS/WAXS and XRF data analysis” section of the Supplementary Materials.

## 3. Results

### 3.1. Radio-Induced Effects on Healthy Treated Rat Brains 

**Figure 2:**
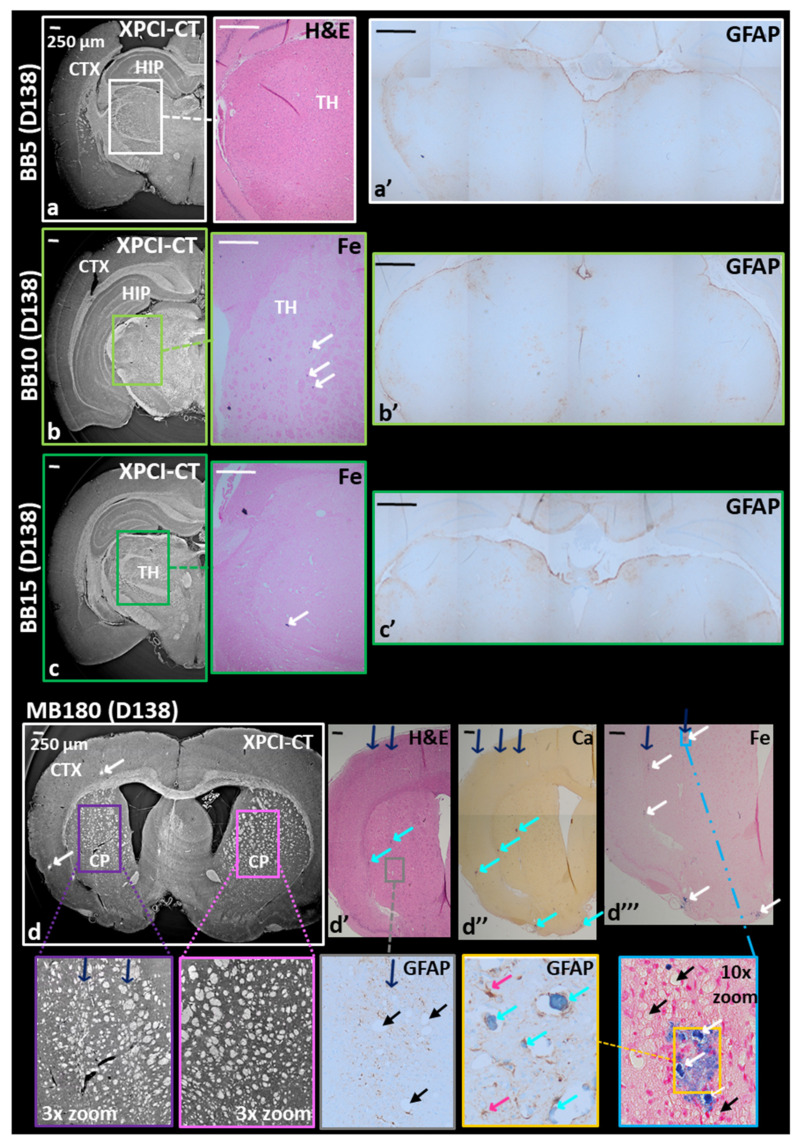
BB and MB180 healthy treated samples. Coronal XPCI-CT images are compared with histology and immunohistochemistry analysis. When the entire slice is not displayed, only the irradiated hemisphere of the brain is reported. (**a**) Shows an XPCI-CT image for the treated side of the BB5 brain reporting no damages in the cortex (CTX), hippocampus (HIP) and thalamus (TH) as confirmed by its H&E and GFAP (**a’**) stained corresponding tissue slices. The BB10 sample is analyzed in (**b**) and (**b’**), where just small Fe deposits are present and marked in blue (see white arrows) in the Fe-stained histology. The GPAF slice reports small reagent uptake in both the hemispheres thus, no RT-induced lesions are revealed. Subfigures (**c**–**c’**) report a BB15 sample analysis where the three different investigations do not show significant pathology: only a small Fe sediment is visible. GFAP staining reveals discrete reactive gliosis in both the hemispheres. MB180 sample display RT-induced scars along the beam path that are visible in XPCI-CT (**d**) especially in the caudate putamen (CP) of the right-side brain and in its 3× zoom together with the left-side counterpart. By this comparison, a reshape in the nervous structures is illustrated along the MB peak delivery areas (indicated by the blue arrows). Subfigures (**d’**–**d‴**) and their insets report the presence of Ca and Fe minerals within the MB-driven scar indicated by cyan and white arrows, respectively together with cell-loss findings displayed as bubbles in the tissue (black arrows) and small astrocytes next to the calcifications. All the images are displayed in the radiological view.

**Figure 3:**
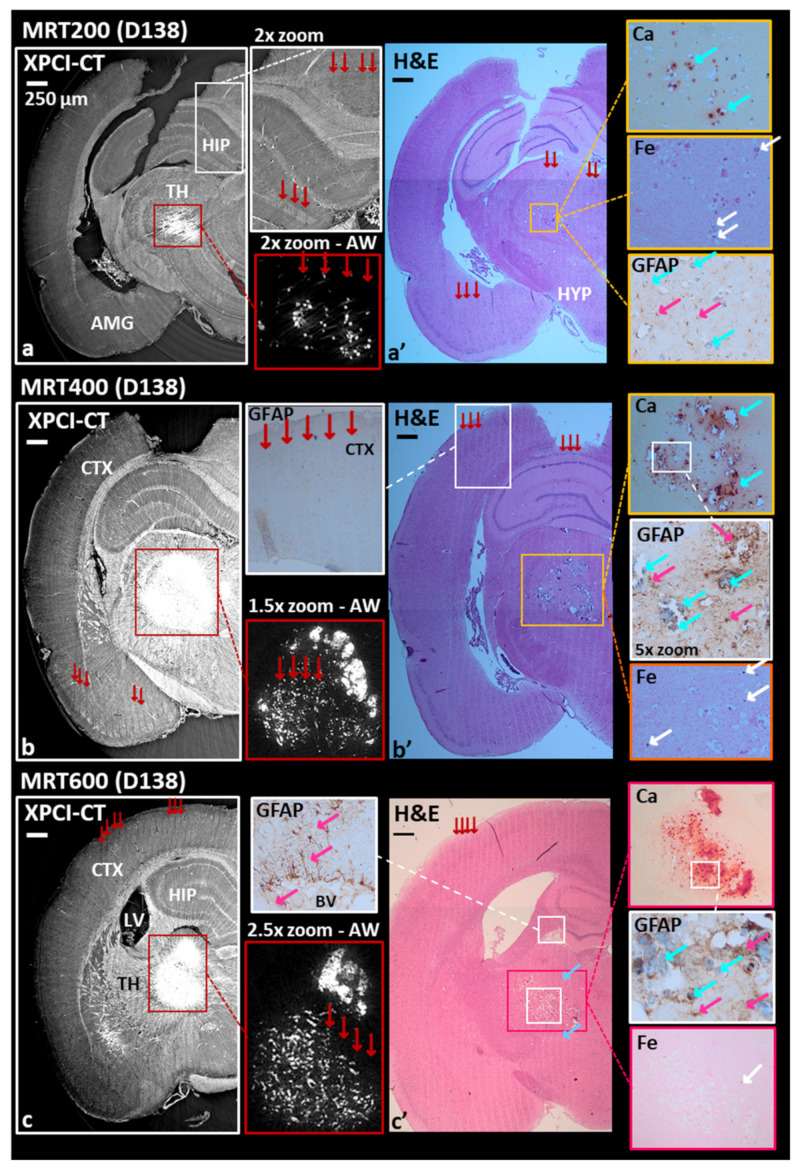
MRT-treated healthy samples: coronal XPCI-CT images compared with histology and immunohistochemistry on the irradiated brain hemisphere. MRT200 sample reports some hyperdense agglomerates in the thalamus (TH) (**a**) that are better visible in the adjusted windowing inset and are recognized as Ca and Fe deposits thanks to the H&E, Alizarin Red and Perl’s Prussian Blue histologies (**a’**). Iron deposits are pointed out by white arrows, while red arrows identify the MRT paths. The GFAP staining of the thalamus area (**a’** inset), shows limited gliosis in two zones. Magenta arrows are for astrocytes and cyan arrows for Ca deposits. MRT paths are visible in both XPCI-CT and histology as tissue ablation in the hippocampus (HIP), thalamus, hypothalamus (HYP) and amygdala (AMG) as visible in the 2× zoom of (**a**) and in (**a’**). MRT400 (**b**) and MRT600 (**c**) samples manifest big cluster of Ca/Fe deposits together with small dot-shaped ones. The different applied stainings reveal a low content in Fe and massive astrogliosis in the deposit surroundings (GFAP staining of (**b’**) and (**c’**)). MRT paths are visible in both XPCI and histologic images. In particular, the cortex (CTX) GFAP inset of (**b’**) demonstrate that no gliosis is induced by MRT irradiation without being entangled to Ca agglomerates.

XPCI-CT coronal images of healthy BB, MB and MRT irradiated brains acquired with a voxel size of 3.25^3^ μm^3^ are presented in Figure 2 and Figure 3 and correlated to histology results. All the images are displayed in the radiologic view and are obtained from samples harvested at D138, which was the decided sacrifice point for all the healthy animals. The main effects on the irradiated tissues caused by the three different treatment modalities are visualized with high contrast and detail. XPCI-CT data are then compared with H&E, Ca, Fe and GFAP stained histological images.

XPCI-CT and histologic images of the BB-treated rat brains are visible in Figure 2a–c, where only the irradiated hemisphere is displayed. In all the irradiated brain regions only histology could reveal the presence of small structures in the BB10 and BB15-treated samples that were recognized as deposits of ferric ion with lateral dimensions of ~30 μm (see the insets of Figure 2b,c). Conversely, the BB5 group does not show any pathological changes. No RT-induced reactive gliosis, as confirmed by GFAP staining, could be observed in any of the three groups. Some brown-marked areas (indicating the presence of astrocytes) are visible in the GFAP histologies of both treated and untreated brain hemispheres, therefore these lesions are not radiation specific and are not necessarily caused by the treatment. 

MB-treated rat brains show evident traces of the dose-delivery geometry. This is clearly visible by comparing the right and left hemispheres of the MB180 brain reported in the coronal XPCI-CT image of Figure 2d and the related 3× zoom insets. The scar produced by the minibeams causes a reshape in the nervous structures in the caudate putamen (CP) as pointed out by blue arrows in the lilac-bordered zoom, to be compared with the homogeneously organized tissue in the pink-bordered inset. Furthermore, along the beam path, hyperdense structures are present as bright spots in XPCI-CT image, as overcolored structures in H&E, and in the Ca and Fe histologic images (Figure 2d–d‴) as indicated by cyan and white arrows (as in all figures), respectively (histologies only show the irradiated hemisphere of the brain). This correlation allows labelling the bright XPCI-CT signal as Ca and Fe deposits. In addition, the MB delivery causes local cell loss and microcystic degeneration of the tissue (black arrows, as in all figures), as visible in the insets of Figure 2d’,d‴. The 10× zoom of Figure 2d‴ shows in detail three Fe mineralizations and some smaller ones in the surroundings. The GFAP staining for the same area, performed on a subsequent slice, shows that Ca deposits (here stained in blue) coexist with Fe ones. In all the images, the blue arrows indicate the MB path direction. Increasing the MB peak dose (e.g., MB350 group), the effects induced on the tissues become more invasive, as shown in Figure S3. The minibeams delivered with a peak dose of 350 Gy causes the complete destruction of the irradiated tissues, which is visible in both XPCI-CT and H&E images. The coronal XPCI-CT (0.7^3^ μm^3^ voxel size) and H&E stained histology insets zoom into the hippocampal lesions (yellow arrows) revealing that a very low content in cells is present producing in some cases small microcystic degeneration in the tissue. Both XPCI-CT and H&E images report the irradiated hemisphere only. The GFAP staining shows a reactive gliosis in the cortex areas corresponding to the valley dose delivery; cell loss is predominant in the peak delivery areas.

By analyzing the XPCI-CT images of MRT-treated animals (Figure 3), tissue micro-ablations, appearing like long micrometer-wide areas with cell losses, are detected in all the irradiated regions of the brain and micro- and macro-deposits of dense materials are visualized, which are identified as Ca and Fe by histologic analysis. These features are shown in Figure 3a–c, where only the irradiated hemispheres are reported. Results obtained on a MRT200-treated brain (Figure 3a–a’) showcase the formation of Ca/Fe deposits in the thalamic area of the right hemisphere along the X-ray microbeam paths (red arrows indicate the MRT delivery direction, as in all figures). On the XPCI-CT image of Figure 3a, the presence of hyperdense, highly absorbing, structures is shown as bright accumulations in the thalamus (TH), while MRT paths are observable in Figure 3a–a’ (XPCI-CT and H&E histology images, respectively) in the hippocampus (HIP), amygdala (AMG), thalamus and hypothalamus (HYP). Thanks to the adjusted windowing (AW) inset of Figure 3a it is noticeable that the bright structures are embedded and appear to be aligned along parallel lines corresponding to the MRT microbeam paths. In Figure 3a it is rather difficult to simultaneously visualize Ca/Fe deposits and the related MRT paths due to the stripe artefacts caused by the abrupt variation of index of refraction between dense deposits and the surrounding soft tissue. The 2× zoom inset of the hippocampus helps recognizing the MRT paths in the XPCI-CT image. The Ca and Fe stained histological slices of the thalamic regions (insets of Figure 3a’) allow for the identification of those deposits as Ca/Fe accumulations, which are revealed as red and blue spots, respectively. For this sample, the GFAP stained inset highlights the presence of some reactive gliosis agglomerates (magenta arrows pointing to astrocytes, as in all figures) within Ca/Fe deposits. In the rat brains of the MRT400 group, large mineral deposits are detected in the thalamus (red-bordered rectangle in the coronal XPCI-CT view in Figure 3b) and they are present both as micro-deposits and as clusters, as it is more clearly highlighted in the AW inset. H&E, Fe, Ca and GFAP histological images (Figure 3b’) attest the presence of an abundant content of Ca with respect to Fe. A massive astrogliosis is visible around the microcalcifications (GFAP 5× zoomed image) while no astrocytes reaction is evident along the MRT paths unless calcifications are present, as demonstrated in the GFAP inset of Figure 3b’, where a portion of a microbeam irradiated cortex (CTX) region is shown. In the MRT600 group, the hyperdense signal on the coronal XPCI-CT image of Figure 3c corresponds again to small deposits and big clusters of granular aggregates (see the AW insert). Those aggregates are mainly composed of Ca, as the different histological stainings also reveal (Figure 3c’ and insets). For this sample, in both XPCI-CT and H&E histology, MRT microbeam paths can be seen crossing the entire hemisphere from the cortex down to the hypothalamus. In the H&E histology (Figure 3c’) the MRT paths are not displayed as straight lines in the thalamus, but they are bent as a result of the tissue deformation due to the Ca deposits formation (light-blue arrows). The two GFAP antibody stained histology images (insets of Figure 3c’) demonstrates again the presence of gliosis in the region around the macrocalcifications and next to a blood vessel (BV) that was probably damaged by the MRT transections.

### 3.2. Effects of Spatially Fractionated Radiotherapy on Glioblastoma-Bearing Animals

#### 3.2.1. Survival Curves

**Figure 4:**
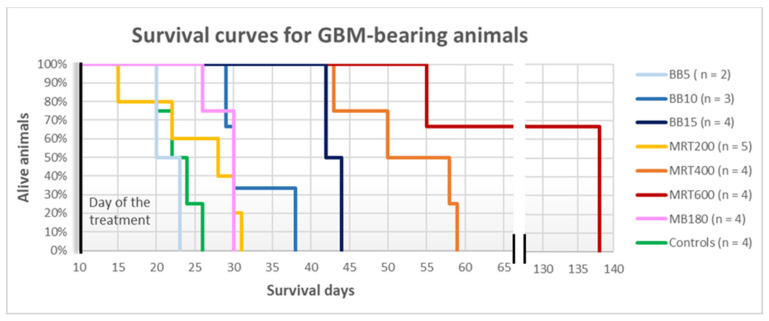
Survival curves of glioblastoma-bearing rats. The curves for the different irradiation groups are given as a percentage of surviving rats as a function of the survival days, counted from the glioblastoma implant day.

The rats belonging to the MB350 groups, both GBM-implanted and healthy, died at D15–16 and are not included in the survival curves. The healthy-treated animals of the MB180 group and of all the BB and MRT groups lived until the programmed end of the experiment, i.e., D138. Therefore, the survival curves reported in Figure 4 only contain data for GBM-bearing animals. Healthy-controls, MRT200 and BB5 groups show a similar survival according to the long-rank-test in a 95% confidence level, resulting in a median survival of 22, 24 and 21 days from the day of GBM implantation, respectively. On the contrary, MRT400 and MRT600 treatments increase the median survival with respect to the untreated group. We obtained a median survival of 52 days for MRT400 and 110 days for the MRT600 group: the last one presents the best survival among the different groups, thus MRT with peak dose of 600 Gy provides the best results in terms of survival in this study. Lastly, the MB180 group shows a median survival of 28 days. 

#### 3.2.2. XPCI-CT: A Multi-Scale Imaging Approach

**Figure 5:**
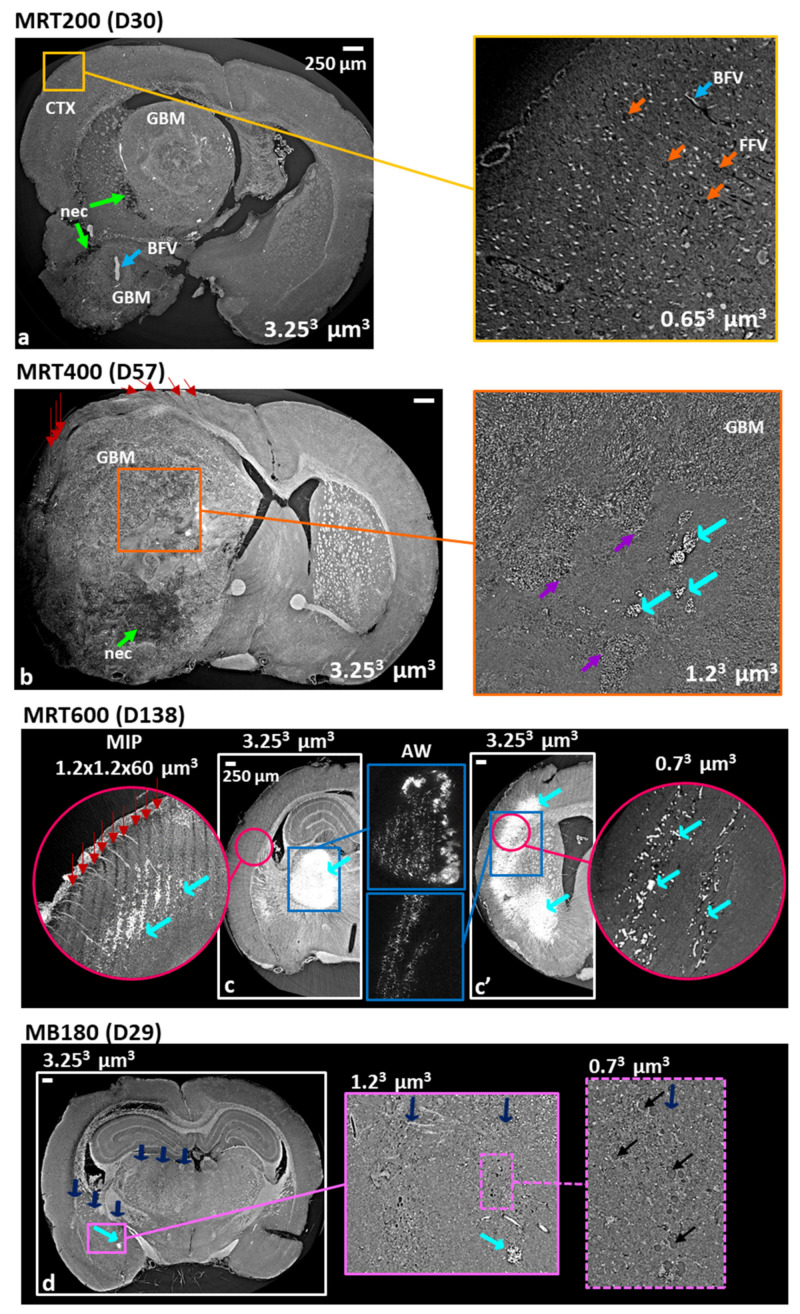
Multi-scale XPCI-CT coronal images of MRT and MB180-treated tumor-bearing brains. The MRT200 sample (**a**) shows great discrimination between healthy and tumor tissue distinguishing necrosis (nec, green arrows) among the GBM structures. A cortex zoom was realized with a 0.7^3^ µm^3^ voxel size setup revealing uniform cell content and no MRT path track. Orange and light-blue arrows indicate formalin (ffv) and blood-filled (bfv) vessel. (**b**) Reports an MRT400-treated sample displaying MRT paths induced tissue ablation (red arrows) and necrosis. Its 1.2^3^ µm^3^ voxel size inset depicts in detail a hypocellular area where calcium deposits are present (cyan arrows). Purple arrows point at the hypocellular area borders. The panels (**c**) and (**c’**) present two different coronal views of the same MRT600-treated brain together with their adjust-windowing insets and high-resolution insets showing in detail that microcalcifications developed along the MRT tracks. The maximum intensity projection (MIP) reveals small deposits that are not visible with 3.25^3^ µm^3^ voxel size. (**d**) Displays in detail the MB180 induced scars together with cells swelling (yellow arrows) and Ca/Fe deposits (cyan arrows) along the minibeam path.

The effects of microbeam and minibeam RTs on glioblastoma-bearing rat brains are shown in Figure 5 by using a multi-scale approach enabling a hierarchical representation of the treated tissues. Coronal XPCI-CT images acquired at different spatial resolutions (i.e., voxel sizes of 3.25^3^, 1.2^3^ and 0.7^3^ µm^3^) are presented. At first, the volume of GBM tumor is well distinguishable against the surrounding healthy tissue as well as the re-organization and disruption of the overall brain anatomy caused by the presence of the tumor (MRT200 specimen in Figure 5a). Necrotic tissue (nec) is displayed with low grey levels (dark area indicated by green arrows) with respect to the other brain tissues and the tumor and blood-filled vessels (BFV) appear as bright features in the XPCI-CT image within the tumor milieu (light-blue arrows). Blood filled vessels appear brighter than the surrounding tissues, as some blood content is still present, since the animals were not perfused and blood cells were not washed out during the sacrifice. The inset of Figure 5a shows an area of the brain cortex of Figure 5a examined with a voxel size of 0.7^3^ µm^3^; in none of these two images the paths of the 200 Gy-peak microbeams are detectable and, overall, for the MRT200-treated brains no sign of the MRT paths is visible in the cortex, that is the MRT entrance area. The structures in the cortex tissue are homogeneously arranged: cells and both formalin (FFV, orange arrows) and blood-filled vessels are clearly recognizable. 

MRT400 brains are showcased in Figure 5b where the tumor is grown replacing almost completely the entire right hemisphere and destroying the healthy structures. Consequently, the brain regions of MRT delivery are replaced by tumor tissue exception made for a small area in the cortex where the signs (cell loss) due to MRT paths are deviated as a result of the tumor growth. Necrotic tissue and intra-tumoral calcifications are visible with a different degree of detail in the 3.25^3^ and 1.2^3^ µm^3^-voxel size-images (Figure 5b and related inset). XPCI-CT image well discriminates the presence of aggregating cells around a hypocellular zone where a denser lump, with an XPCI-CT signal compatible with Ca (see cyan arrows), appears too. Purple arrows point at the borders of this hypocellular area, while cyan ones indicate the agglomerates. The multi-scale XPCI-CT images of an MRT600-treated brain acquired using voxel sizes of 3.25^3^, 1.2^3^ and 0.7^3^ µm^3^ show that small mineralizations can be discriminated by using the highest spatial resolutions, and that a 3.25^3^ µm^3^ voxel size is not sufficient, instead, to separate individual microcalcifications. Figure 5c,c’ are two coronal XPCI-CT slices of the same sample illustrating different anatomical areas of the irradiated hemisphere where both MRT paths and large Ca/Fe agglomerates are evident (see the AW insets). The 1.2^3^ µm^3^ voxel size (pink-bordered) inset is a MIP of 50 subsequent XPCI-CT images (corresponding approximately to a 60 µm thick slice) around the slice shown in Figure 5c. This MIP image highlights the presence of small agglomerates, compatible with the microcalcifications found in other anatomical regions, that are not detectable when using a 3.25^3^ µm^3^ voxel size. Both the high-resolution insets of Figure 5c,c’ display micro agglomerates developing along the MRT peak dose paths. Finally, XPCI-CT coronal images of a tumor-bearing rat brain treated with MB180 are shown in Figure 5d. By applying the multi-scale XPCI-CT approach, it is possible to depict the MB-induced scars (blue arrows) with Ca agglomerates and microcystic-like cell loss regions (black arrows).

### 3.3. Quantification and 3D Rendering of Radiotherapy Effects

**Figure 6:**
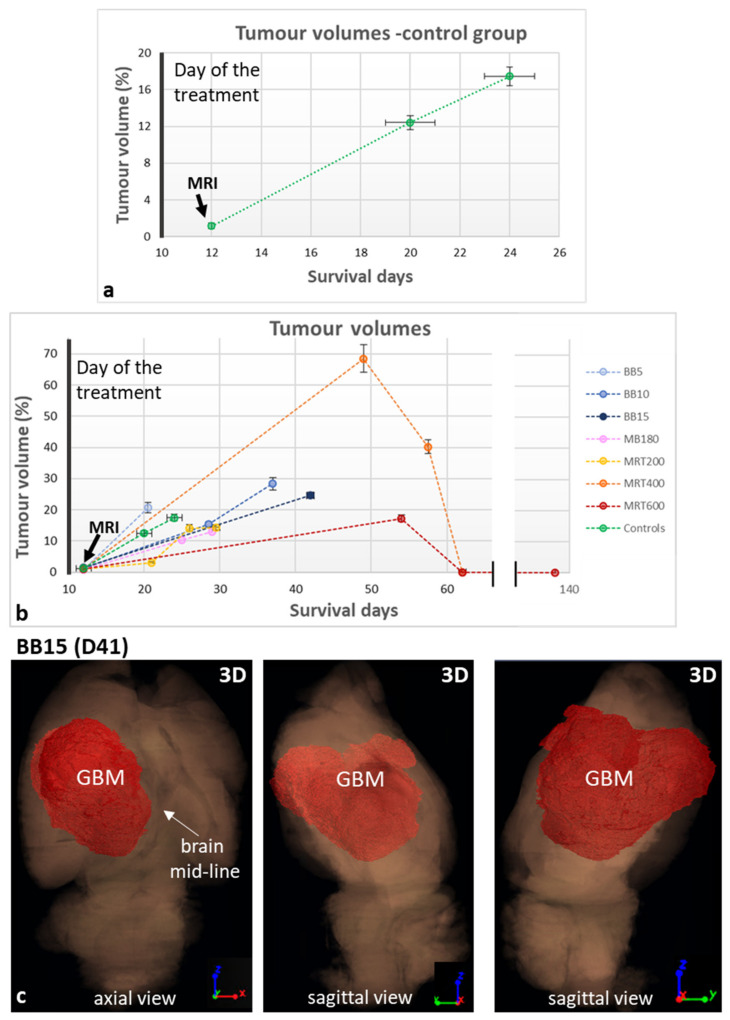
Quantification of tumor volumes for the GBM-control group (**a**) and all the groups involved in the study (**b**) as a function of the survival days which are counted starting from the tumoral cells implantation day (D0). All the values are extracted from XPCI-CT images but the one at day 12, which is in-vivo MRI-based. Tumor volumes are given as a percentage of the entire brain volume; values of animals belonging to the same group with survivals within three days are averaged and the mean values are reported in the graphs. (**c**) Reports three different orientations (axial and two different sagittal views) of the 3D rendering of a BB15 irradiated rat brain showing how the tumor develops into the brain volume shifting the brain mid-line. The full organ dataset is rendered in semi-transparency while the tumor is colored in red and co-registered as a solid mass.

**Figure 7:**
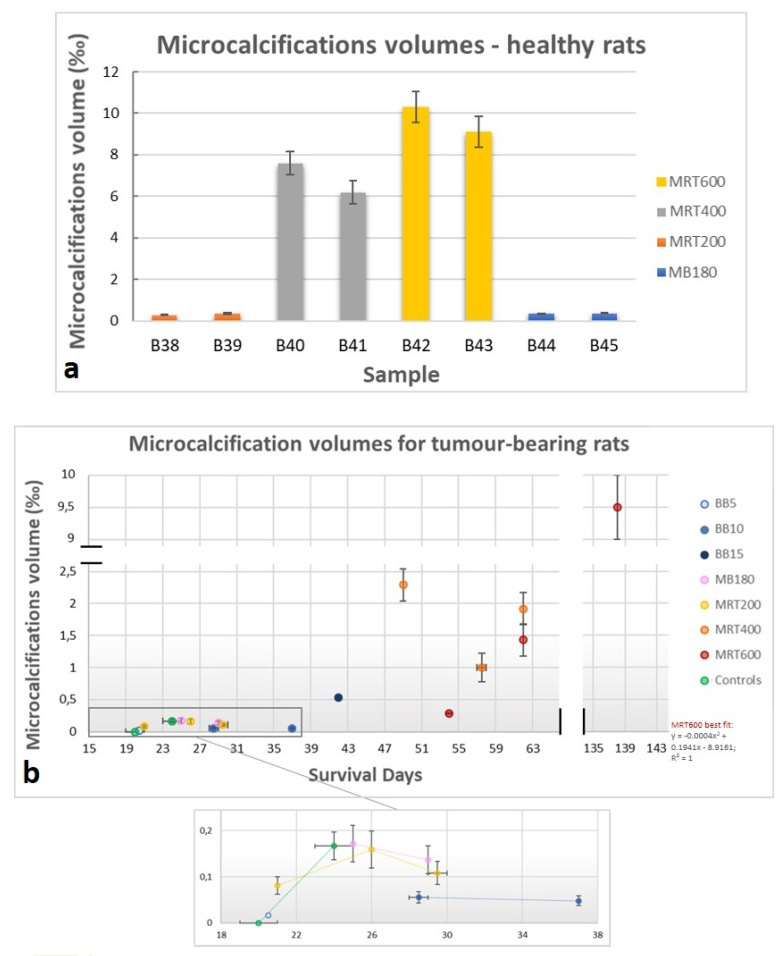
Graphs of the microcalcifications content in healthy irradiated rats sacrificed at D138 (**a**) and in GBM-bearing animals as a function of the survival days (**b**). Values are normalized over the field of irradiation. The zoom in (**b**) better discriminates the values obtained before D39.

Results of the characterization and quantification study of tumor and microcalcifications for all the irradiation groups are reported in Figure 6 and Figure 7. Tumor volumes graphs report both the volumes assessed with MRI and XPCI-CT according to the results of the compatibility test performed as explained in the Materials and Methods section. The compatibility test between MRI and XPCI-CT tumor volume measurements is reported in Table S1 showing that, overall, the MRI and XPCI-CT segmentation procedures are compatible on six out of the eight considered samples. Furthermore, if the averaged values (last raw) of all the computed parameters are considered, MRI- and XPCI-CT-based tumor volumes are overall within the compatibility. As a result, the values of tumor volumes obtained from in-vivo MRI and ex vivo XPCI-CT data can be displayed in the same plot.

The GBM-control group (Figure 6a) as well the BB groups (Figure 6b) show a linear trend for the tumor development over time. Furthermore, as noticeable in Figure 6b, the MRT200 and MB180 groups have values of tumor volumes similar to those of the BB5 and GBM-control groups. The MRT400 group presents the largest tumor volumes among the different groups and the MRT600-treated brains show the smallest tumor volumes achieved within this study. For animals surviving more than 60 days, no tumor residue is detected on the 3.25^3^ µm^3^-voxel-size XPCI-CT images. Nevertheless, some residual GBM infiltrations are detected with GFAP stained immunohistochemistry, as can be seen in Figure S4, meaning that no complete tumor sterilization has been achieved. Here, the tumor is indicated by high cellularity and nuclear pleomorphism, i.e., variability of size, shape and chromatin density of the nuclei.

As a case in point, Figure 6c reports the 3D rendering of one tumor-bearing brain sample of the BB15 group. Three different views are reported (one axial and two sagittal views). The brain volume is rendered in a semi-transparent mode while the glioblastoma is colored in red and rendered as a solid mass. The three different views allow identifying how the irregular GBM shape has developed within the organ, shifting the brain mid-line (axial view). Other tumor 3D renderings are presented in Figure S5 showing how the glioblastoma has grown in MRT200 and MB180-treated brains.

**Figure 8:**
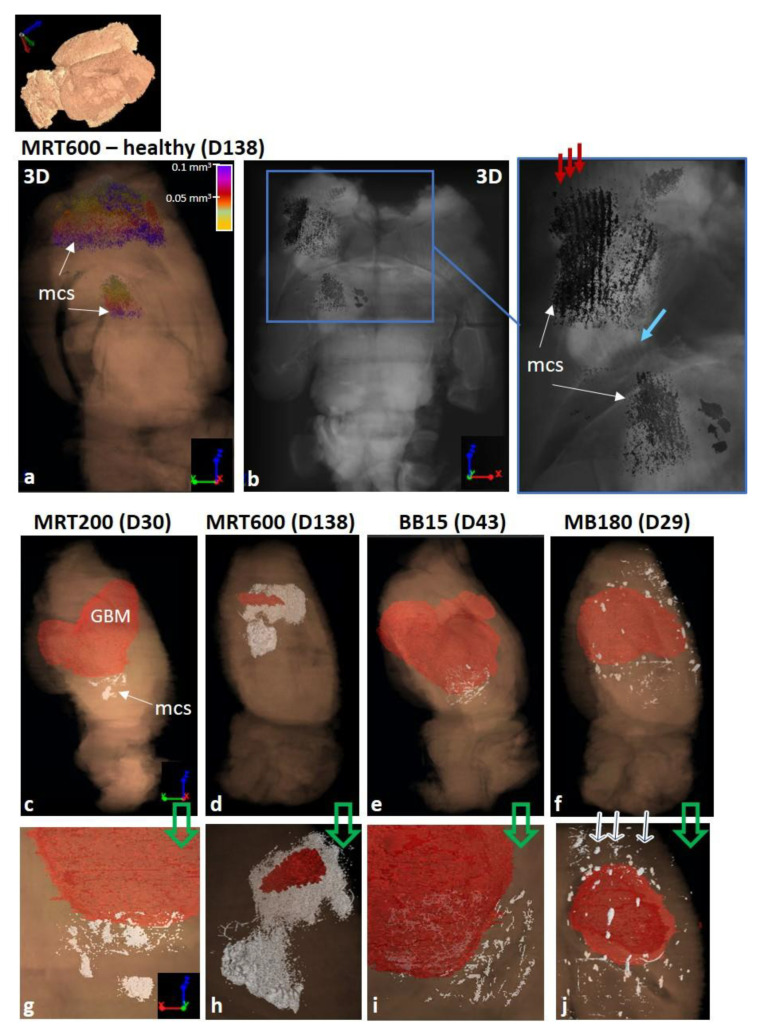
(**a**,**b**) Three-dimensional (3D) images of the distribution of microcalcifications (mcs) for a healthy MRT600-treated sample in a sagittal and axial view, respectively. The (**d**) zoom enhances that microcalcifications are distributed along the MRT paths (red arrows) and the green arrow points out that the MRT paths are bended along their track. (**c**–**f**) The tumor and microcalcifications 3D rendering, in a sagittal view, within the entire brain organ for an MRT200, MRT600, BB15 and MB180 - irradiated sample respectively. (**g**–**j**) The zoomed rendering, in axial view, of (**c**–**j**) respectively. All tumor and brain volumes are presented in semi-transparency while microcalcifications are co-registered to the other volumes as solid white deposits.

As for the assessment of the microcalcifications’ volume, all the healthy animals treated with MRT and MB180 show microcalcifications within the irradiated tissues, as reported in the histogram in Figure 7a. The MB180 and MRT200 groups have comparable total volumes of microcalcifications; for the MRT-treated groups it increases as the peak dose levels rise. All the tumor-bearing animals, exception made for two GBM-controls, show microcalcifications; the total volume of microcalcifications vs the survival days are reported in the plot in Figure 7b. The total volume of microcalcifications in the MRT200, BB5, BB10, MB180 and GBM-control animals present similar values and trends over time, while the MRT400 group has no specific trend and the MRT600 volumes have a quadratic growth over time. A 3D representation of the distribution of the microcalcifications (indicated as mcs in the figures) within the whole brain and within the irradiated tissues for an MRT600-treated healthy brain are shown in Figure 8a,b, respectively. Figure 8a shows the sagittal view of the whole 3D rendered brain, displayed in semi-transparent modality, and the co-registered segmented microcalcifications volume. Here it is possible to appreciate how microcalcifications are formed within the brain and that they agglomerate in the thalamus, caudate putamen, frontal, parietal and orbital cortex. Furthermore, in the axial view of Figure 8b and its inset, the grey-scale 3D rendering of both the organ and microcalcifications allow identifying the location of the structures with respect to the beam paths, indicated by the red arrows. The light-blue arrow points to a region of the tissue where the tacks of the MRT microbeam paths appear “bended” in the proximity of the Ca/Fe deposits. As a comparison, the 3D rendering of an MRT200-treated healthy brain is presented in Figure S6. In this case, the microcalcifications only develop in the thalamus as non-clustered agglomerates of different dimensions.

The coexistence of tumor and microcalcifications within the treated brains is shown, for different irradiation geometries, in Figure 8c–j. Images in Figure 8c–f report the sagittal view of the 3D rendering of an MRT200, MRT600, BB15 and MB180 samples respectively, while 3D renderings in Figure 8g–j are zoomed views of the regions with microcalcifications on an axial orientation, for the same samples. The brain and the tumor volumes are rendered with the same color code as in the previous figures, while microcalcifications are here all depicted in white. As for the healthy case, MRT200 samples only show microcalcifications in the thalamus; Figure 8g displays the deposits next to the tumor borders, similarly to BB15. The MRT600-treated sample, if compared with the MRT200 one, shows more extended calcifications in the area from the thalamus to the cortex. The zoom of Figure 8h allows discriminating both micro- and macro-deposits. Lastly, in the MB180 sample (Figure 8f,j) microcalcifications are clearly arranged along the MB paths (see the arrows) along the entire irradiation field. 

### 3.4. SAXS/WAXS and XRF Study of Microcalcifications

**Figure 9:**
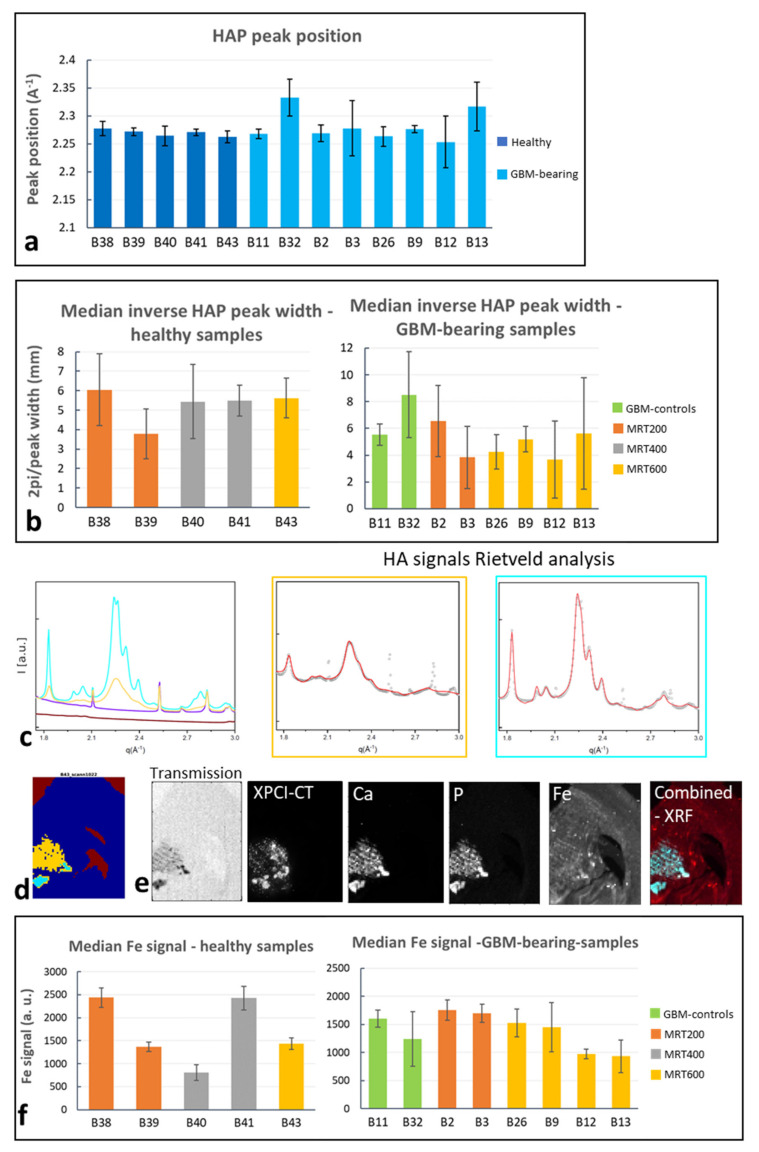
SAXS/WAXS and XRF data analysis for controls and MRT-treated samples. (**a**) Peak position graph of the samples showing hydroxyapatite (HAP) as crystallized phase for the Ca content. (**b**) Inverse peak width for healthy and GBM-bearing MRT-treated brains. The reported value is the median over all the detected signals within the q-range from 2.20 Å^−1^ to 2.34 Å^−1^. (**c**–**e**) Signal classification, showcase the presence of two different populations of HAP within the same sample, which is revealed by the two WAXS signals, the yellow and cyan one, in (**c**) that are also displayed with their Rietveld analysis. Here, the crystalline domains evaluated along the 002 direction are 11.7 and 27.0 nm. The colored micrograph (**d**) helps in identifying the sample areas associated with the detected WAXS signals. (**e**) The micrographs obtained for the same specimen, for transmission, XPCI-CT, Ca, P, Fe and combined XRF signals. (**f**) Mean XRF Fe signal for all the available samples. Fe signal was only studied in the image pixels where a relevant XRF Ca signal is detected.

SAXS/WAXS and XRF analysis enabled the structural and chemical classification of microcalcifications for MRT-treated brain samples. In all the investigated specimens showing crystallized Ca, WAXS identified the Ca content as hydroxyapatite (HAP) crystals. The HAP peak position, evaluated for the most intense peak (q~2.28 Å^−1^) is reported in Figure 9a, showing that all these samples, which are the ones with a crystalline Ca content, include HAP. For the same samples, the median value of the HAP crystalline domains (∝ 2π/peak width) evaluated along the most intense peak, are reported in Figure 9b, where the median value over all the detected signals is reported. Figure 9b shows that, for both healthy and GBM-bearing MRT-treated brains, no specific trend as a function of the delivered MRT peak dose is recognizable and all the samples exhibit the formation of HAP crystals with similar domain sizes. It must be noted that the GBM-bearing MRT600 values are from animals that were sacrificed at different time points, while the others are all sacrificed at the last time point. Overall, the graphs of Figure 9b report the median values, but large error bars (+/− one standard deviation) reveal that, in some cases, different HAP WAXS profiles are distinguished, corresponding to distinct HAP crystalline domains, i.e., peaks have different FWHM. As a showcase, in Figure 9c the most representative WAXS signals found within a healthy MRT600-treated sample are reported. Yellow and cyan profiles correspond to HAP and their Rietveld analysis and crystalline domain evaluation along the [2] direction, which is the (002) peak at q = 1.83 Å^−1^, are reported in the caption. The colored 2D sample map (Figure 9d) helps in identifying the HAP regions and the sub-areas corresponding to the two HAP-WAXS profiles (same color code in Figure 9c,d). It is noticeable that the largest crystalline domain value is associated with low signal in the transmission and high XPCI-CT, Ca, Fe and P XRF signals (Figure 9e), all expressed in arbitrary units. The “Combined-XRF” image reports the combined Fe, Ca and P XRF signals in RGB scale. Thus, the areas in white exhibit the coexistence of the three elements, while cyan areas are rich in Ca and P; the red Fe background present in the entire slice is due to the used stainless-steel microtome blade. A similar behavior was found in other samples: SAXS/WAXS and XRF collected data are reported in Figure S7 for a GBM-control sample, as a further example. 

The Fe median signal was extracted for each sample as explained in the Materials and Methods section, and is reported in the graphs of Figure 9f. For the healthy-treated samples, no specific trend is visible, and a large intra-group variability is shown, while for the GBM-bearing brain all values are compatible to each other. In these plots, some samples show large error values indicating that the Fe signals detected in the different pixels is spread over a large interval of values. By looking at the GBM-bearing MRT-600-treated samples, a decrease in the mean value for the Fe signal is visible for the animals sacrificed at D138 (samples B12–B13).

## 4. Discussion

### 4.1. Effects of Treated Healthy Rat Brains

The potential of XPCI-CT as a tool for investigating the effects induced by radiotherapy has been shown analyzing rat brains treated with both standard and spatially-fractionated RTs. First, the study was performed on healthy brains to access how the brain responds to the different RT protocols. All the delivered BB irradiations (using 5, 10 and 15 Gy) do not determine visible pathological signs or tissue alterations; histological images only reveal a few Fe deposits (see Figure 2). The presence of astrocyte excess in the BB-treated brains is likely due to rat age (D138 for all the animals) and do not represent a pathological state as confirmed by a symmetric GFAP uptake in the two hemispheres (Figure 2). 

MRT-treated samples show more pronounced effects that appear as tissue ablations corresponding to the peak delivery areas together with microcalcifications agglomerates, as visible in Figure 3. During the treatment, the following brain areas were irradiated: neocortex, hippocampus, thalamus, hypothalamus, caudate putamen, frontal, parietal and orbital cortex. The MRT-induced ablations are visible in all these areas for the MRT400 and MRT600 groups, while the MRT200-treated animals exhibit ablations mainly in the thalamus and hypothalamus (as showcased in Figure 2). MRT-induced tissue ablations were already seen in Barbone et al. 2018 and Bouchet et al. 2016 [25,53], but no preferential effect with the applied dose and the anatomical brain area has been reported. Nonetheless, MRT transection preserve the overall neuroanatomy and some neurons are still visible within the microbeams peak delivery area, as confirmed by histology. Microcalcifications are visible in a small amount in the MRT200-irradiated samples, and the amount of deposits increases by increasing the peak dose value, as visible in the plot of Figure 7a. Overall, microcalcifications are observable as old (i.e., advanced stage) and well-organized lesions, probably caused by local micro-bleeding from blood vessels (see the dedicated paragraph). The MRT200 microcalcifications are mainly found in the thalamus (Figure S6) while for the MRT400 and MRT600 groups they also appear in other anatomical regions (for instance in the caudate putamen and orbitofrontal cortex) accompanied by massive astrogliosis (Figure 3 and Figure 8). The fact that microcalcifications are only present in specific areas of the brain suggests a different radio-sensitivity of the different brain anatomical areas, as discussed later in the paragraph dedicated to the 3D-based information. This is also confirmed by the presence of bended microbeam paths next to Ca/Fe agglomerates in healthy samples (Figure 3c’ and Figure 8b): slow-down of metabolism causes deposits and thus, the MRT bending occurs at the interface with an area with more accelerated metabolism. 

MB-treated rat brains present pronounced scar induced by the MB passage, which are characterized by Ca and Fe deposits, in a smaller amount with respect to all the MRT cases, and by low cell density areas (Figure 2d–d‴ and Figure S3) that are well distinguishable with both histology and high-resolution XPCI-CT (Figure 5d). This latter type of lesion derives from a degradation of the tissue and a neuronal band interruption that are typical signs of old-occurred localized lesions that did not result in a complete tissue destruction nor in a repaired necrosis. MB and MRT groups showcase how the dose-volume effect determines very different outcomes in spatially fractionated RTs depending on the size of the field of irradiation. Limited to the investigated cases, microbeams are well tolerated by the brain tissues for radiation doses up to 600 Gy of peak, while minibeams cause important damages (see Figure S3) even if the peak and valley doses are both lower than in the MRT600 case. Lastly, it has to be considered that the MB350 valley dose is compatible with the uniformly delivered dose in the BB15 case, where no substantial effects are found. Thus, the used protocol for MB350 animals represents a dose limit that should not be exceeded when planning the minibeam RT with the used spacing. This evidence is corroborated by the fact that all the MB350-treated animals, both healthy and tumor-bearing, died at D15-16.

### 4.2. Effects on GBM-Bearing Rat Brains

The main visible effects of the RTs on tumor-bearing animals concerns the tumor control, i.e., the regression or progression of the tumoral entity, to be interpreted together with the analysis of survival curves and the occurrence of microcalcifications. The high sensitivity of XPCI-CT with respect to the nervous tissue structures allows for an optimal GBM discrimination, as previously demonstrated in the literature [25,54,55]. This capability is evident in Figure 5a–b where the GBM tissue and necrosis are appreciable, as in [56], at different length scales enabling the establishment of a solid and precise pipeline for tumor segmentation that can be also applied to other animal models. The implemented ilastik-based segmentation tool exploits the 3D nature of the XPCI-CT data providing, isotropically, greater precision in tumor segmentation with respect to the one achievable with histology or MRI. Indeed, histologic-based tumor quantification is intrinsically 2D (the thickness of the cut slices determines the spatial resolution in the third dimension), and the MRI-based one has the drawback of being based on low spatial resolution data, especially in the third dimension where is limited by the inter-space between two subsequent slices [8,30,53]. Thanks to the implemented segmentation procedure, it was possible to build the graphs of Figure 6a,b reporting the tumor development over time. The first plot reports a tumor volume trend for the GBM-controls compatible with the linear growth found in [53,57] and the second show the computed tumor volume for all the different groups. As in [53], the tumor volumes for BBs and MRT200 groups have an upward trend as a function of time and overall, for the BB groups, the median survival is increased as the delivered dose increases. The MRT400 group shows large values of the tumor volumes and are not in accordance with the available literature. Probably, this is due to the low statistics and to the intra-animal variability in reacting to treatment since those values represent data from a small number of animals. The MRT600 group shows the best tumor control achieved in this study and, for animals surviving more than 60 days, no traces of GMB are detected by inspection with 3.25^3^ µm^3^ voxel size XPCI-CT. However, GFAP stained histologies reveals small GBM traces in all these samples (Figure S4), meaning that these tumor infiltrations are below the 3.25^3^ µm^3^ voxel size detectability limit. Figure 7d,h showcase in 3D the MRT600 induced tumor shrinkage and thus regression.

### 4.3. Microcalcification Study

By analyzing the microcalcifications content in the different samples, it is possible to see that those deposits are both radio- and tumor-induced. Healthy MB180- and MRT-treated animals exhibit pure radio-induced accumulations (Figure 7a), GBM-controls display pure tumor-induced mineralizations and GBM-bearing treated animals show the presence of both types of HAP crystals (Figure 7b). On the contrary, healthy-controls do not show any Ca/Fe accumulation. Thus, the radio- and tumor-induced microcalcifications are caused by two different sources but do not show significant differences from a chemical and structural point of view (see SAXS/WAXS and XRF results in Figure 9). In the literature, the presence of microcalcifications has been demonstrated in MRT-treated GBM-bearing and healthy brains [15,28] and on healthy MB-treated brains [13] with different geometrical parameters or applied radiation doses. Although in this study the segmentation was performed on the full-organ 3.25^3^ µm^3^ voxel size CT datasets, the multi-scale approach applied in targeted regions allows for a better identification of small deposits, as shown in Figure 5. 

Microcalcifications are a well-known post-irradiation effect. Ca deposits are classified as a late effect of cranial irradiation in childhood [58]. This effect is known as mineralizing microangiopathy, an autoimmune reaction localized to the irradiated area that is associated with vasculitis and hyalinization [59]. Thus, hypersensitivity of the vessels after irradiation produces vascular damages with hypoxia that results in Ca mineralization. Mineralizing microangiopathy found after child and adult cranial irradiations is mainly present in the basal ganglia and is asymptomatic in most cases [60]. These observations strengthen the XPCI-CT based evidence of microcalcifications mainly occurring in the thalamus and caudate putamen that are strictly connected or part of the basal ganglia, respectively (Figure 3 and Figure 8). XPCI-CT enables the full-organ 3D analysis with great detail and sensitivity allowing the investigation of RT-induced effects and their localization. Histological examination of the mineralizing microangiopathy deposits showed a strong positive stain for Ca and a weak positive stain for Fe [61], as also seen in our cases (histologies of Figure 2 and Figure 3). Furthermore, brain irradiation was found to cause microbleeding in survivors irradiated during childhood [62], which is likely to increase during the follow-up, and is classified as a small vessel disease [63]. Thus, microbleeding and mineralizing microangiopathy can be seen as the responsible for microcalcifications formation in the irradiated animals of our study.

For the first time an in-depth analysis of the chemical and crystalline nature of the microcalcifications was realized by means of SAXS/WAXS and XRF tools. Interestingly, many MRT-treated samples showed a crystallized phase of Ca that was identified with HAP crystals by means of the WAXS analysis (Figure 9a). The microcalcifications content for healthy MRT-treated samples show an increasing trend with the peak dose (Figure 7a), but no visible trend for their crystalline domain values or XRF Fe intensity signal is retrievable. It is worth to note that in Figure 9b the plotted values of crystalline domains are the typical (median) values obtained for each sample, but the coexistence of different HAP populations in the same specimen is detected (Figure 9c and Figure S7) suggesting a heterogeneous formation of HAP deposits within the brain tissue. All samples containing HAP crystals exhibit the coexistence of Ca, Fe and P in the same sites (Figure 9e and Figure S7). Nevertheless, some GBM-bearing samples were found to contain Fe (from XRF measurements) and no crystallized HAP (as from WAXS analysis) in the region where there is a XPCI-CT signal compatible with that of microcalcifications. Probably, an X-ray amorphous phase of HAP is present, thus corroborating the evidence that no specific values of crystalline domains can be associated with each MRT group. In the literature, the presence of HAP calcifications on humans is reported in various pathological cases (see [51,64,65]), where mineralizations are associated with cells injury, explaining the radio-induced calcifications or with apoptosis and necrosis, which cause a pathological release of high concentrations of calcium and phosphate that can explain the detected GBM-induced microcalcifications.

### 4.4. A Full 3D Characterization and Quantification of RT-Induced Effects

The 3D rendering of the distribution of the tumor and microcalcifications within the study organ shown in Figure 6 and Figure 8 displays how XPCI-CT enables an accurate volumetric visualization of anatomical structures and pathological states. From the tumor volume rendering it is possible to see how the tumor develops inside the full brain for the three different radiotherapy protocols (BB, MRT and MB) shifting, in some cases, the brain midline and consequently displacing the healthy surrounding tissues. 

As for the microcalcifications 3D rendering, in Figure 8 and Figure S6, it is possible to have a visual and qualitative assessment of the different radio-sensitivity of brain anatomical regions depending on the applied RT protocol. As it can be seen in Figure 8e,i, BB cases show microcalcifications in the thalamic area if only associated with tumor presence. This could be explained considering that tumor areas are characterized by a fragile vasculature where microbleeding is more likely to happen and thus Ca/Fe deposits can build in. Healthy BB-treated animals do not show evident clusters of Ca/Fe and only isolated deposits are present (see Figure 2). MB-treated brains do not display evident differences in radio-sensitivity for microcalcifications formation. Clusters of mineralizations are present along the full FOI reproducing the MB path with discontinuity (see Figure 8f,j). Healthy-treated brains show about three times more Ca/Fe deposits with respect to the GBM-bearing ones, exception made for the MRT600 group. This is probably due to the tumor growth within the organ and to metabolic processes that remodel the presence of microcalcifications and their formation processes within the brain with respect to the healthy cases. The MRT-treated brains have a very different content in microcalcifications depending on the delivered dose (see Figure 8 and Figure S6). Nevertheless, the thalamus is the most sensitive brain area to X-ray MRT irradiation (as also confirmed by the aforementioned studies on mineralizing microangiopathy): microcalcifications are present in all the MRT-treated brains (200–600 Gy of peak dose) regardless the peak dose. Analyzing the distribution of microcalcifications on MRT400 and MRT600-treated animals, they appear also to be visible in the caudate putamen and in some cortex areas. Furthermore, it is noticeable that only MRT200-treated animals do not show clustered deposits. At the best of our knowledge, no previous work reported experimental 3D results on the induced effects of RTs and thus on the different radio-sensitivity within the different brain areas. 

### 4.5. Limitations of the Study

The main limitation of this study is related to the low statistics in terms of number of animals available for each RT group. This is justified by the fact that in the first phase of the project the aim was to test the sensitivity of the approach to different irradiation conditions (different groups). The survival curves are reported to show the curing power of the different RT protocols on the GBM-bearing irradiated animals, but they were not presumed to be statistically significant at this stage of the study. Furthermore, SAXS/WAXS and XRF analyses were only performed on one representative tissue slice per each sample. Additional experiments have been planned to increase the sample numbers and the statistic relevance of the quantitative assessments.

### 4.6. Translational Aspects of the Research

*In-vivo* XPCI-CT applications exist and are already set up for particular target organs and purposes such as breast mammography and CT [66,67,68], joint cartilage imaging for the diagnosis of arthritis [69,70] and lung diseases detection [71,72,73] putting the basis for exploiting XPCI-CT as an in-vivo RT follow-up technique. Aspects that need to be addressed and optimized in view of the in-vivo application of XPCI-CT are the dose issue related to the use of ionizing radiation and the linked questions of the applicable spatial resolution and scan duration. The doses currently delivered when imaging post mortem soft tissues are strongly dependent on the chosen voxel size, among the different experimental parameters. As an example, for a single CT performed at ESRF-ID17 with a monochromatic beam of 35 keV, the dose delivered to an excised soft-tissue sample (e.g., a rat brain) is about 150 Gy with a voxel size of 3.25^3^ μm^3^, while for voxel sizes of 6^3^–8^3^ μm^3^ the dose ranges from hundreds of mGy to few Gy, which is in line with the delivered doses in conventional micro-CT full-animal scans [74,75]. The dose can be further lowered if, e.g., iterative or machine learning methods are used for CT reconstruction [76,77]. 

Another aspect to be taken into account is associated to the physiological motions of living organisms. The smaller the used voxel size, the more sensitive is the technique to the small movements of the animal and to motions within the animal’s body (breathing, heartbeat, etc.) and the higher is the risk of image artefacts that can jeopardize the quality of the resulting images. Overall, voxel sizes of the order of 6^3^-10^3^ μm^3^ are a good candidate for XPCI-CT in-vivo applications.

Additional limitations may arise when the soft tissue to be examined is embedded within a bony structure, as in the case of in-vivo brain imaging. The presence of the skull may impair the visibility of the soft cerebral structures. The limits encountered in imaging complex organisms due to the co-presence of both soft and hard tissues can be mitigated by the use of multi-material phase retrieval algorithms as demonstrated by recently published works [78]. Previous studies by our group already demonstrated that XPCI-CT may facilitate a more complete evaluation of complex samples and organisms by providing concurrent comprehensive information about soft and hard tissue [79,80].

As far as it concerns the spatially fractionated RTs, due to the micrometric width of the single beamlet, very short irradiation time (high dose rates) are needed to obtain a precise MRT dose distribution within the treated tissue: any movement of the target would cause the smearing of the MRT lateral dose profile. X-ray beams issued at third and fourth generation synchrotron facilities are particularly adapted for this treatment because of their inherent high collimation (quasi-parallel X-rays) and fluxes (i.e., high dose rates). These requirements are less stringent for the MB technique. Indeed, MB was developed as an alternative to MRT to (partially) overcome these difficulties [13,81]: small movements occurring during irradiations will not merge the millimeter-distant MB beamlets and lower doses with respect to the MRT case are needed. Over the past years, technical efforts have been also addressed for the development of alternative X-ray sources for MRT and MB for translation into clinical practice. As an example, line-focus X-ray tubes are being considered as microbeam source and Monte Carlo simulations have demonstrated they have a great potential as a radiation source for clinical application [82], of MRT. With this respect it is also important to mention recent studies in which in-vitro MRT treatments performed with laboratory sources at conventional dose rates enhanced an increased tumor cell sensitivity [83,84]. 

## 5. Conclusions

This study proves that the XPCI-CT imaging technique is well suited for visualizing ex-vivo, with a label-free, 3D full organ approach the neuroanatomy of irradiated brains and side effects after radiotherapy. The effects of standard and novel radiotherapies on both healthy and tumor-bearing rat brains are visualized with high sensitivity, quantified and classified by a multi-technique approach. The comparison of XPCI-CT with histology, immunohistochemistry, SAXS/WAXS and XRF analyses enabled the morphological, structural and chemical categorization of radio-induced effects on brain tissues. 

This is the first study that compares X-ray BB, MRT and MB treatment protocols, provides an accurate 3D visualization and quantification of tumor and microcalcifications volumes and demonstrates a non-uniform radio-sensitivity of the different brain areas. Microcalcifications are also identified, from a multi-technique approach, as HAP with a coexistence of Fe, Ca and P. Supplementary investigations are necessary to study if the microcalcifications crystalline structures varies depending on the brain area in which they develop and to compare the presented results with behavioral studies. The qualitative and quantitative methodologies here presented are of high value for an accurate and precise evaluation of the efficacy of treatments. Additional experimental sessions are planned to improve the statistical significance of the results of this study and to extend the analysis of the evolution over time of the radio-induced effects of these novel radiotherapies.

Overall, this study proves that XPCI-CT is a valuable imaging technique for a post mortem follow-up of full organs at high resolution. It also puts the basis for channeling studies of in-vivo applications for monitoring RT effects.

## Figures and Tables

**Table 1:** Irradiation parameters for healthy and GBM-bearing animals. The sacrifice day column is referred to the GBM-bearing groups. The sacrifice day for healthy animals was set to D138. * D26, 41 and 61 where fixed as sacrifice points to study the tumor and microcalcifications evolution.

RT Group	Peak Dose (Gy)	Valley Dose (Gy)	Beam Width (μm)	c-t-c Distance (μm)	Sacrifice Day for GBM-Bearing Animals
BB5	5	--	--	--	20–23
BB10	10	--	--	--	29–38
BB15	15	--	--	--	42–44
MRT200	200	7.7	50	200	15–31
MRT400	400	15.3	50	200	43–59
MRT600	600	23.0	50	200	26, 41, 61, 55–138 *
MB180	180	7.2	500	1000	26–30
MB350	350	14.0	500	1000	15–16
Controls	--	--	--	--	20–26

## Data Availability

The datasets supporting the conclusions of this article are available in the European Synchrotron Radiation Facility repository and will be shared upon request to the corresponding author.

## Supplementary Materials

The following are available online at https://www.mdpi.com/article/10.3390/cancers13194953/s1, [85,86,87,88,89,90,91,92]. Figure S1: Ilastik segmentation workflow. The segmentation procedure is applied on a set of XPCI-CT images undergoing the procedure here displayed for one single image. Original 16bit images (**1**) are used to segment the tumor out and are subsequently reshaped for reducing the dataset size (**2**). A variance filter is applied with a radius of 4 pixels (**3**). A homogeneous image without edges would be displayed in black, while in the presence of edges a brighter signal is displayed. For enhancing the tumor borders, the original image is divided by the filtered one (**4**). Thanks to the carving tool of the ilastik software, the object of interest and the background can be labelled by the user (**5**). Based on that, the software identifies the object to be segmented. For a full dataset, it is sufficient to label the 10% of the available slices, the full-volume segmentation will be done by interpolation. After the tumor is correctly identified (**6**), binary images are produced (**7**) and the tumor volume can be quantified by the Analyze Particles Fiji plugin (**8**). This volume can also be rendered in 3D via the software VGStudio MAX 4.3 together with the original brain dataset (**9**). Figure S2: Ilastik segmentation validation. For four samples the comparison of GFAP histology staining (first column) with the XPCI-CT images (second column) and the segment tumor (third column) is given. One slice for each sample is reported. Good agreement is shown for three samples (**a**–**a”**), (**b**–**b”**) and (**c**–**c”**), while for the images (**d**–**d”**) a good matching is visible for the solid tumor mass, but the ilastik segmentation overestimates the GBM infiltrations shown in the magnified insets. Figure S3: XPCI-CT multi-scale approach for a MB350-trated brain and comparison with histology and immunohistochemistry. The minibeam delivery caused a complete tissue ablation: see the XPCI-CT image and the H&E histology (white and blue arrows point at the minibeam paths). The GFAP cortex (CTX) zoom shows reactive gliosis in the minibeam valleys while in peak delivery area cell loss is predominant, as seen in the XPCI-CT and H&E hippocampal (HIP) zooms. Black arrows indicate the cell swelling induced by radiation. Figure S4: Coronal XPCI-CT and GFAP immunohistochemistry images comparison for GBM-bearing MRT600-treated samples sacrificed 69 (**a**,**b**) and 138 (**c**,**d**) days after the tumor implant. Only the implanted hemisphere is shown. (**a**) The GFAP stained coronal histology and (**b**) the respective XPCI-CT image obtained at similar magnification. The insets show, at the maximum available magnification, the residual GBM: the GFAP staining reveal the presence of necrosis (blue bordered inset) and hypercellularity (light-blue bordered box), while the two XPCI-CT insets (yellow borders), obtained with two windowings, do not show abnormal structures due to the numerous streak artefacts. (**c**,**d**) The GFAP and XPCI-CT coronal slices for another GBM-bearing rat brain where XPCI cannot detect any residual tumor (see yellow bordered insets), while immunohistochemistry reveals the presence of residual GBM infiltrations with increased cellularity and nuclear pleomorphism (blue bordered inset). Figure S5: Three-dimensional (3D) tumor rendering of an MRT200 (**a**)–(**b**) and MB180 (**c**)–(**d**) treated rat brain. The full brain dataset is displayed in semi-transparency while the tumor volume is obtained from ilastik segmentation and rendered as a solid red volume. The axial and sagittal views allow understanding, in 3D, of how the tumor has developed within the brain. Figure S6: Microcalcifications 3D rendering for an MRT200-treated healthy rat brain. The brain XPCI-CT dataset is given as semi-transparent volume while the macrocalcifications (mcs) are reported as colored solid deposits of dimensions according to the legend. This 3D rendering showcases that MRT200-induced microcalcifications are mainly present in the thalamus. Figure S7: SAXS/WAXS and fluorescence data for a GBM-bearing control brain. (**a**) The four WAXS profiles detected in the sample shown in (**c**) on a transmission image. In (**a**) the Rietveld analysis of the HAP signals (red and cyan profiles) is also displayed in the colored bordered boxes. (**b**) The sample image in which every pixel is colored according to which WAXS profile is detected in it. (**d**)–(**i**) The 2D micrographs of the XPCI-CT signal, HAP map, Ca, P, Fe and combined fluorescence signals for this GBM-control sample. Table S1. Compatibility study of the MRI- and XPCI-CT-based tumour volumes (TV) for the MB350 group. The table reports the animal name, the survival counted from the day of glioblastoma implant, XPCI-CT and MRI tumour volumes normalized to the full brain volume with errors and the compatibility evaluation of MRI-XPCI-CT values together with the statistical indicators P(t) and the confidence level (C.L.). MRI-XPCI-CT tumour volumes with C.L.>5% are considered compatible each other. The last raw reports the average of the columns.
