# Supplementary material for: A Multi-Scale and Multi-Technique Approach for the Characterization of the Effects of Spatially Fractionated X-ray Radiation Therapies in a Preclinical Model
Source: Cancers (Basel). 2021 Oct 1;13(19):4953. doi: 10.3390/cancers13194953 (PMC8507698; doi:10.3390/cancers13194953)
Supplement: Supplementary file 1 [file cancers-13-04953-s001.zip › cancers-1350933-supplementary.pdf]

Supplementary materials

Tumor Segmentation

First, the tumor area was cropped to reduce the data size; secondly, a variance filter normalization was applied to reduce possible streak artefacts due to discontinuities of the index of refraction (as hyperdense structures are present) and to better enhance the tumor borders. For every image, a variance radius of 4 was used leading to a 9x9 square kernel on which, for each pixel, the variance (σ) is calculated according to $\sigma^2 = \frac{1}{n}\sum_{i=1}^{n}(u_i - \overline{u})^2$ where n = 81, u_i is the intensity of the ith pixel and $\overline{u}$ the median intensity [85]. This implies that homogeneous tissues are displayed in black while, in the presence of edges, a brighter signal appears as shown in Figure S6. Then, for every slice a normalization of the original slice with the filtered one was performed (i.e., the original slice was divided by its variance filtered version) allowing to clearly distinguish the tumor volume (displayed in black) from the surrounding tissue (characterized by lighter grey values). The final dataset is segmented with the ilastik carving method (watershed-based [86]) producing binary images from which the tumor volume was quantified by using the Analyze particles Fiji plugin.

Histologic Procedures

Tissue slices were transferred on glass slides (SuperFrost PLUS, Thermo Scientific, Waltham, Massachusetts, United States of America) dewaxed and rehydrated following standard protocols: preheating to 60 °C for 10′, Neo Clear xylene substitute (Merk KgaA, Darmstadt, Germany) 2× or 10′, Ethanol graded series (100%, 96%, 90%, 80% and 70%, 1× each for 5′), water 1× 5′). Dedicated staining protocols followed.

H&E staining were performed following standard protocols. Shortly, after dewaxing and rehydration, Mayer´s Haemalaun solution (Merk KgaA) was used for nuclear staining (5 min) and Eosin G solution (0.5%; Roth, Karlsruhe, Germany) was used for cytoplasmic staining (12 sec), followed by 80% Ethanol (3x for 5′), Ethanol graded series (90%, 96% and 100%, 1× each for 3′), Neo Clear (2× 5′). Tissue was finally covered using Neo-Mount (Merk KgaA).

Alizarin Red staining was obtained following a protocol described in the literature [87], while Fe deposits were stained putting each slice in Perls' Prussian Blue solution for 30 min, rinsing in distilled water for 5 min, then a 0.1% Nuclear Fast Red solution was used for 10 min, ascending alcohol series and xylene. The two latter protocols allow staining Ca and Fe deposits with red and blue pigments, respectively.

After dewaxing and antigen demasking (microwave irradiation at 600 W, 0.1 M Citrate Buffer, pH6) slides were incubated with the rabbit anti-GFAP antibody (clone E4L7M, Cell Signalling, Frankfurt am Main, Germany; 1:100) over night at 4°C. Tissue slides were further processed using the EnVision System HRP (DAB) kit (Agilent, Frankfurt am Main, Germany) according to the manufacturer´s instructions. Tissue slices were counterstained with Mayers Haemalaun (Merk KgaA) and covered with Kaiser´s Gycerine Gelatine (Merk KgaA).

SAXS/WAXS and XRF Data Analysis

To identify characteristic features for each sample, the azimuthally averaged 1D WAXS and SAXS patterns were statistically analyzed using a signal-classification method [88] to extract the least-correlated profiles. This allows efficient screening of a large data-set and extraction of few profiles for further analysis. In particular, the few 1D WAXS profiles, selected by this classification procedure, were first indexed by means of the crystallographic software QUALX 2.0 [89] to identify the crystalline structure that can explain all the diffraction peaks in the pattern. This first analysis allowed us to identify the Ca5(PO4)3(OH)-HAP [90], as the unique crystallographic structure which best describes the WAXS patterns. We then proceeded with fitting the patterns with the whole-profile Rietveld-based program FULLPROF [91] which was used to prove the qualitative hypothesis on the chemical-physical origin of crystalline structure as the origin of the measured WAXS patterns and to quantify the HAP crystalline domain size along precise crystallographic directions. Precisely, we proceeded as follows: the crystalline structure composition of the sample, namely the atomic positions, the space group (P 63/m), the hexagonal unit cell sizes $a = b = 9.465$ Å and $c = 6.9095$ Å, was provided to the program and refined. The inhomogeneous peak broadening of the diffraction peaks was described by a modified Scherrer formula (phenomenological model based on spherical harmonics), with the instrumental resolution function (IRF), evaluated by fitting the diffraction pattern of a LaB6 NIST standard, recorded under the same experimental conditions, and provided to the program [92].

Going back to the high throughput data analysis, one can determine pixel by pixel the relative abundance of the selected profiles, or selected peaks, in the imaged sample area. Here, for the WAXS data, a single peak analysis has been performed around q = 2.28 Å^{-1}, from 2.20 Å^{-1} to 2.34 Å^{-1}, the location of the most intense diffraction peak characteristic for HAP, which was described as a single Gaussian peak whose position, width and intensity has been determined on a q^{-n} background. This background has been chosen as it fits universally both the often q^{-4} to q^{-1} signal in in SAXS, that

is for the peak-analysis regarded as background, and the often locally constant background in WAXS. Once the single peak fitting procedure was repeated for each pixel of the microscopy and for each sample, characteristic values were extracted by calculating for each sample the median peak position and width across all pixels with a reliably detectable HAP peak signal at $q = 2.28$ Å^{-1}. Furthermore, the standard deviation has been calculated to characterize the spread of the distribution. For the XRF analysis, the total intensity in the three energy ranges characteristic for the elements Fe, Ca, and P has been determined for each pixel. Also here, characteristic values have been determined by calculating the median and the standard deviation and its spread. As an alternative method to the WAXS signal for locating HAP calcifications, the XRF Ca signal can be used. To define the threshold for a relevant Ca signal, we calculated for each sample the median Ca signal for all pixels and then the median of these values over all samples, and multiplied it by three. We checked via the standard deviations in non-calcified areas that this threshold is typically beyond the signal occurring due to variations of the Ca signal observed outside calcifications. For each sample, among the pixels with a Ca signal above this threshold, the median P, Ca, and Fe XRF signal has been calculated, as typical values for the calcified areas. To specify the spread of these XRF signals, two types of standard deviations have been calculated. The standard deviation over all pixels exhibiting a Ca signal above threshold and the one calculated over the central 50%, when sorting the XRF signal within that area for P, Ca, or Fe by the size of the signal. This standard deviation of the central 50% is mainly characteristic for the spread of the values that determine the specified median value, whereas a normal standard deviation is taking into account outliers with equal weight.

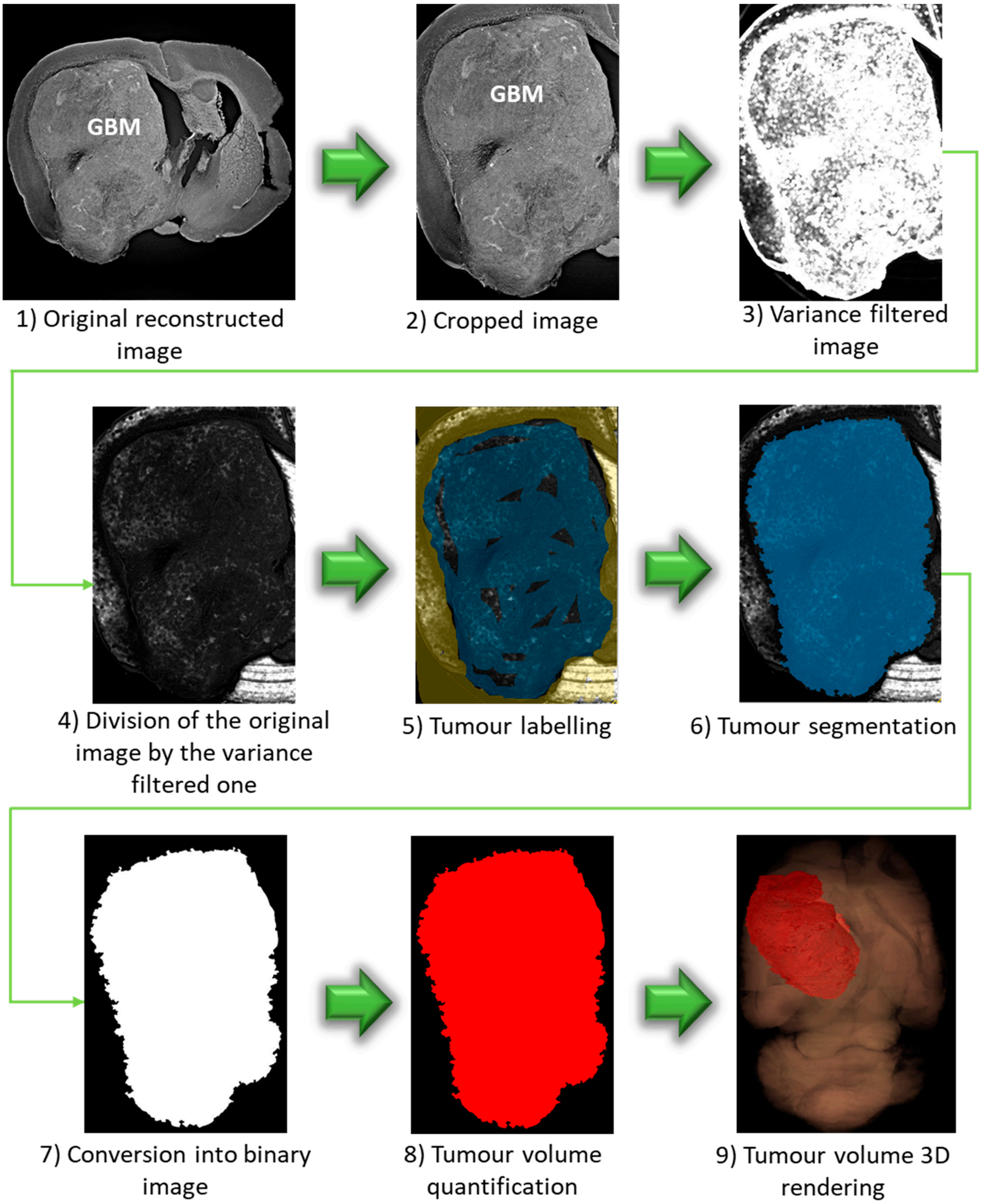

Figure S1. Ilastik segmentation workflow. The segmentation procedure is applied on a set of XPCI-CT images undergoing the procedure here displayed for one single image. Original 16bit images (**1**) are used to segment the tumor out and are subsequently reshaped for reducing the dataset size (**2**). A variance filter is applied with a radius of 4 pixels (**3**): a homogeneous image without edges would be displayed in black, while in the presence of edges a brighter signal is displayed. For enhancing the tumor borders, the original image is divided by the filtered one (**4**). Thanks to the carving tool of the ilastik software, the object of interest and the background can be labelled by the user (**5**). Based on that, the software identifies the object to be segmented. For a full dataset, it is sufficient to label the 10% of the available slices, the full-volume segmentation will be done by interpolation. After the tumor is correctly identified (**6**), binary images are produced (**7**) and the tumor volume can be quantified by the Analyze Particles Fiji plugin (**8**). This volume can also be rendered in 3D via the software VGStudio MAX 4.3 together with the original brain dataset (**9**).

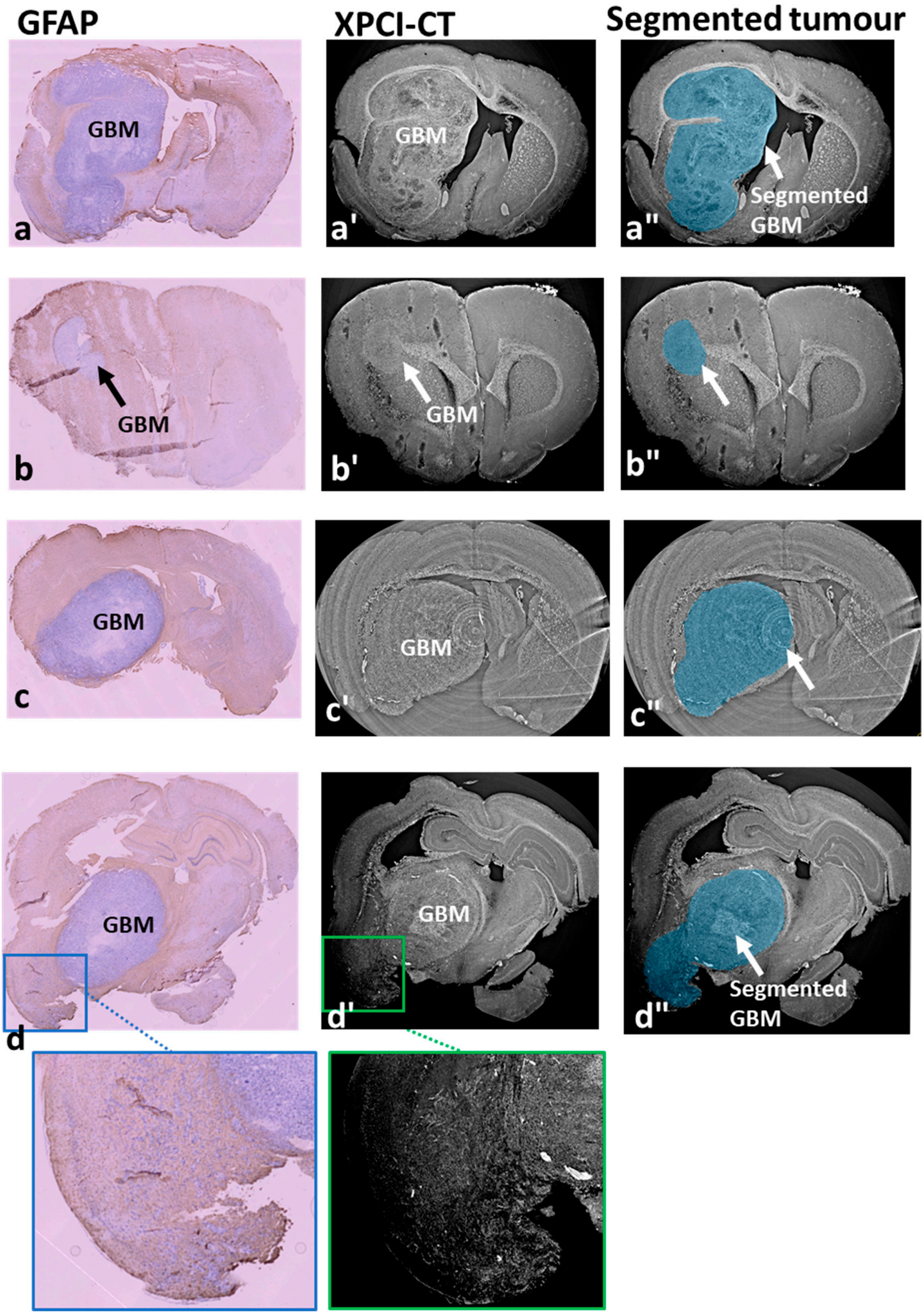

Figure S2. Ilastik segmentation validation. For four samples the comparison of GFAP histology staining (first column) with the XPCI-CT images (second column) and the segment tumor (third column) is given. One slice for each sample is reported. Good agreement is shown for three samples (**a**–**a**″), (**b**–**b**″) and (**c**–**c**″), while for the images (**d**–**d**″) a good matching is visible for the solid tumor mass, but the ilastik segmentation overestimates the GBM infiltrations shown in the magnified insets.

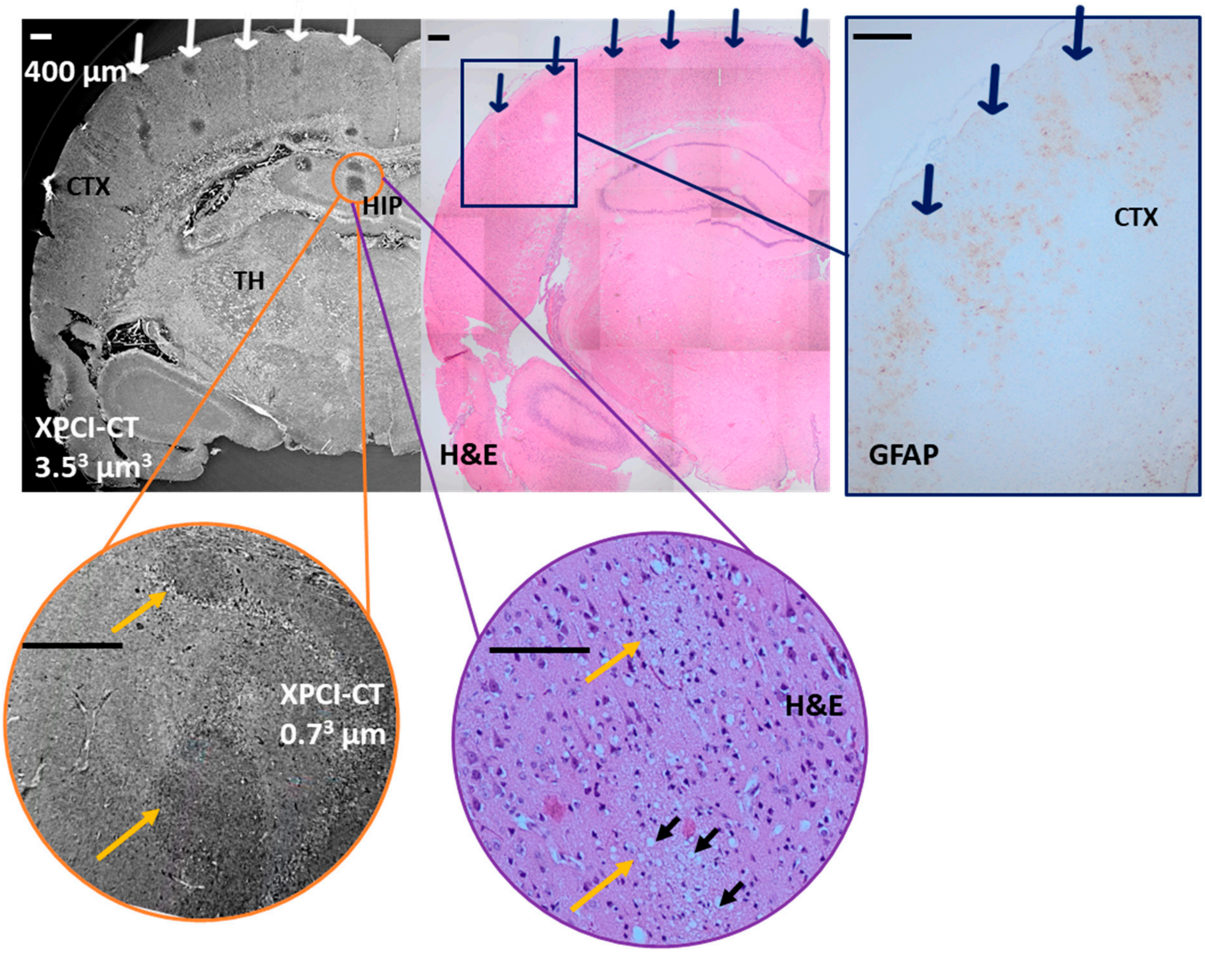

Figure S3. XPCI-CT multi-scale approach for a MB350-trated brain and comparison with histology and immunohistochemistry. The minibeam delivery caused a complete tissue ablation: see the XPCI-CT image and the H&E histology (white and blue arrows point at the minibeam paths). The GFAP cortex (CTX) zoom shows reactive gliosis in the minibeam valleys while in peak delivery area cell loss is predominant, as seen in the XPCI-CT and H&E hippocampal (HIP) zooms. Black arrows indicate the cell swelling induced by radiation.

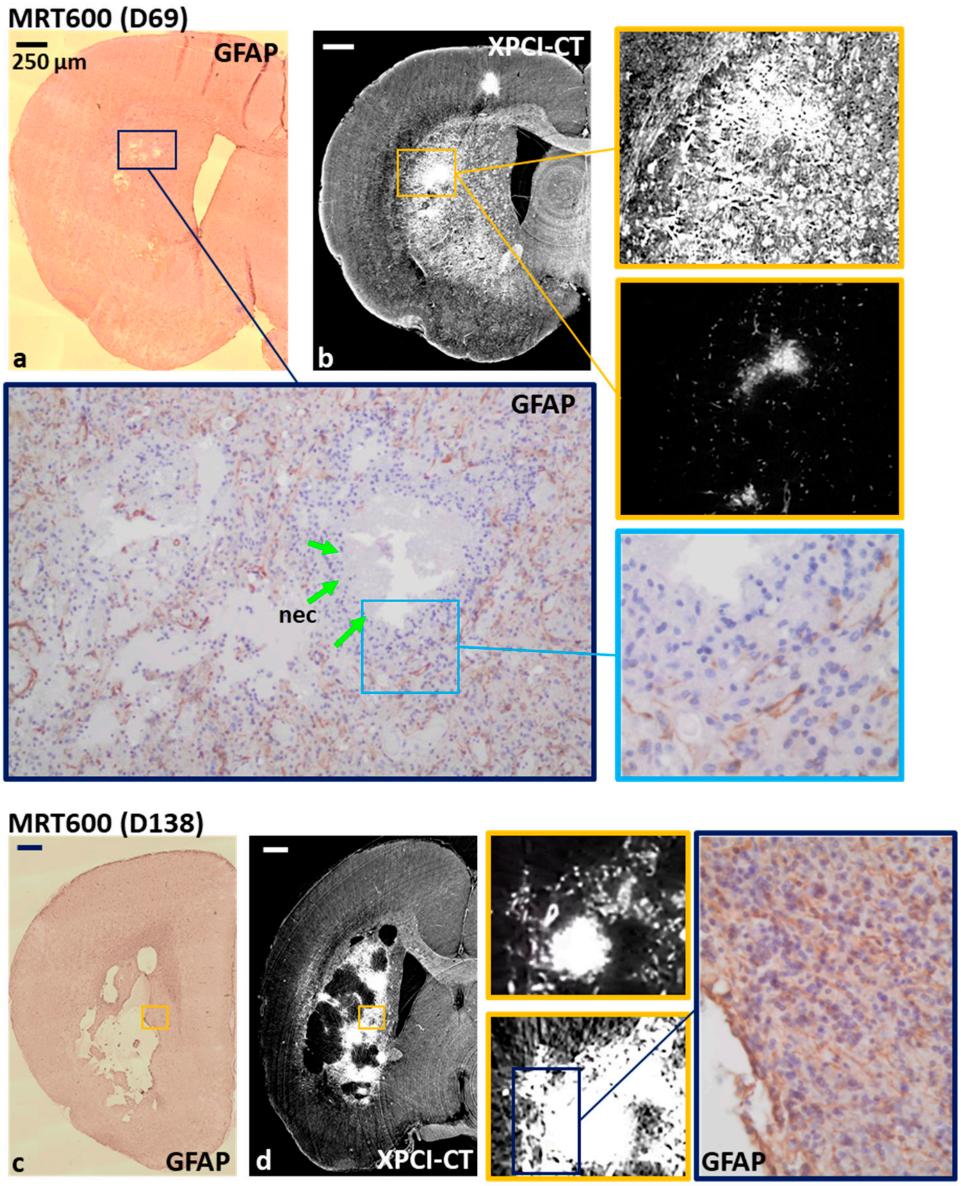

Figure S4. Coronal XPCI-CT and GFAP immunohistochemistry images comparison for GBM-bearing MRT600-treated samples sacrificed 69 (**a**,**b**) and 138 (**c**,**d**) days after the tumor implant. Only the implanted hemisphere is shown. (**a**) The GFAP stained coronal histology and (b) the respective XPCI-CT image obtained at similar magnification. The insets show, at the maximum available magnification, the residual GBM: the GFAP staining reveal the presence of necrosis (blue bordered inset) and hyper-cellularity (light-blue bordered box), while the two XPCI-CT insets (yellow borders), obtained with two windowings, do not show abnormal structures due to the numerous streak artefacts. (**c**,**d**) The GFAP and XPCI-CT coronal slices for another GBM-bearing rat brain where XPCI cannot detect any residual tumor (see yellow bordered insets), while immunohistochemistry reveals the presence of residual GBM infiltrations with increased cellularity and nuclear pleomorphism (blue bordered inset).

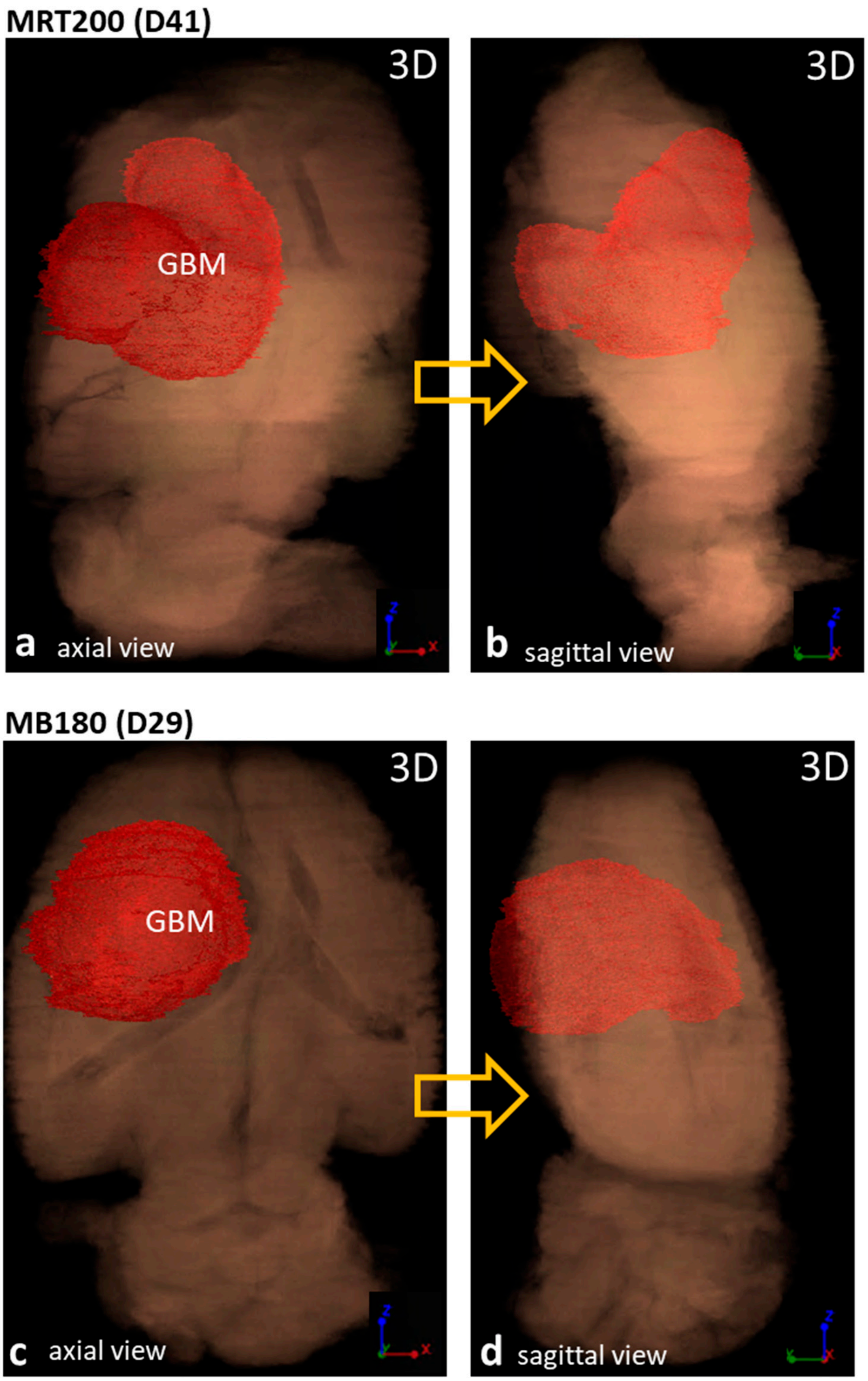

Figure S5. Three-dimensional (3D) tumor rendering of an MRT200 (**a**)–(**b**) and MB180 (**c**)–(**d**) treated rat brain. The full brain dataset is displayed in semi-transparency while the tumor volume is obtained from ilastik segmentation and rendered as a solid red volume. The axial and sagittal views allow understanding, in 3D, of how the tumor has developed within the brain.

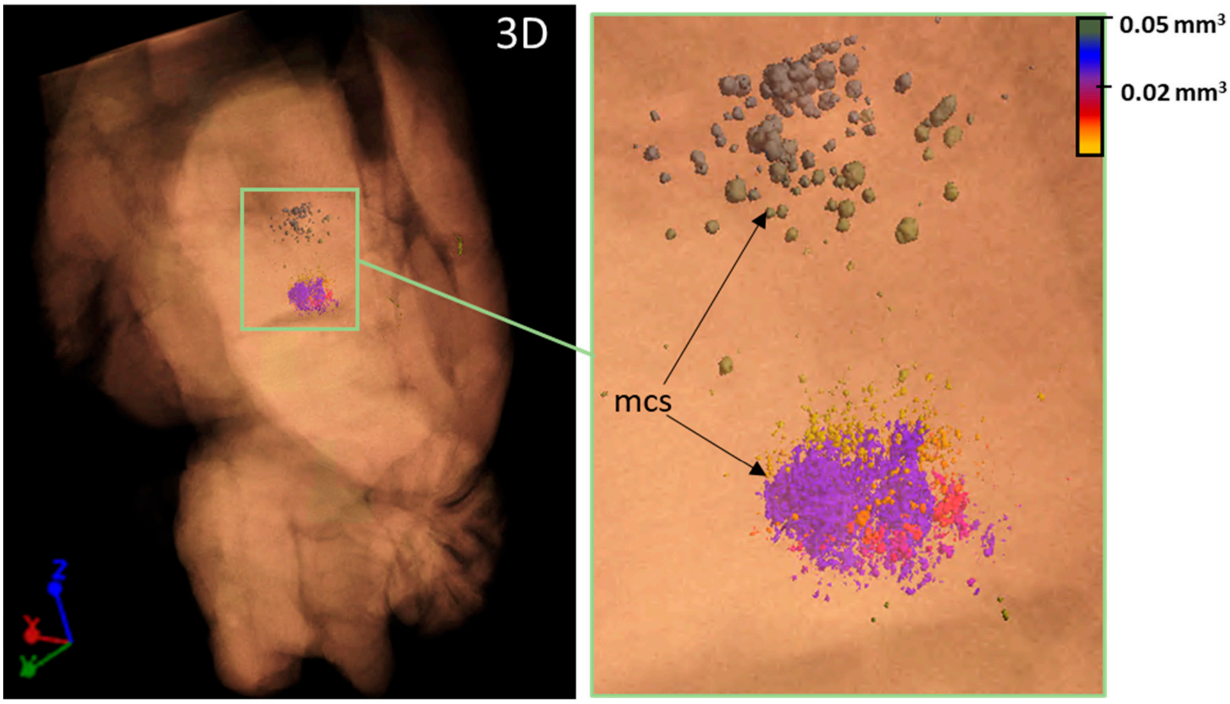

Figure S6. Microcalcifications 3D rendering for an MRT200-treated healthy rat brain. The brain XPCI-CT dataset is given as semi-transparent volume while the macrocalcifications (mcs) are reported as colored solid deposits of dimensions according to the legend. This 3D rendering showcases that MRT200-induced microcalcifications are mainly present in the thalamus.

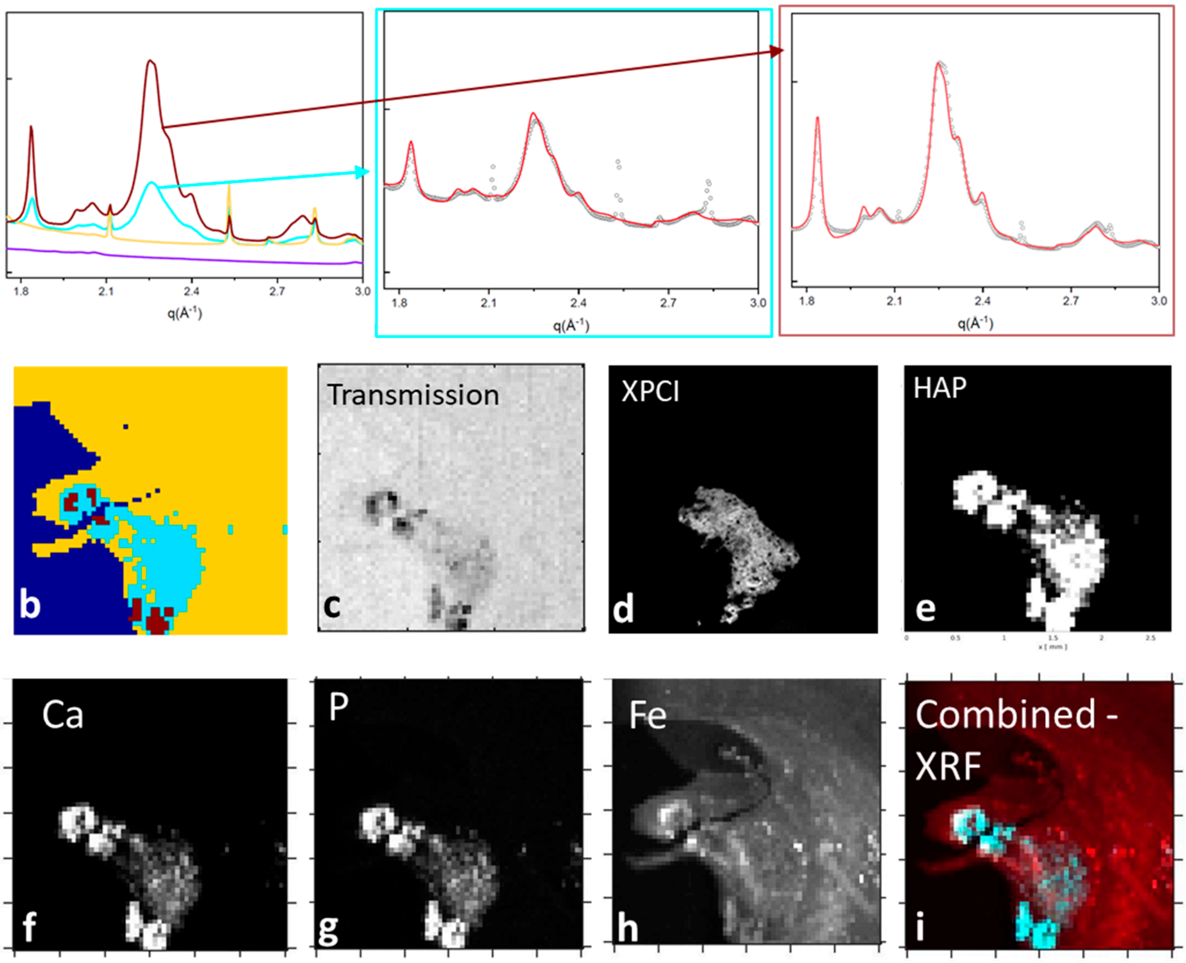

Figure S7. SAXS/WAXS and fluorescence data for a GBM-bearing control brain. (**a**) The four WAXS profiles detected in the sample shown in (**c**) on a transmission image. In (**a**) the Rietveld analysis of the HAP signals (red and cyan profiles) is also

displayed in the colored bordered boxes. (**b**) The sample image in which every pixel is colored according to which WAXS profile is detected in it. (**d**)–(**i**) The 2D micrographs of the XPCI-CT signal, HAP map, Ca, P, Fe and combined fluorescence signals for this GBM-control sample.

Table S1. Compatibility study of the MRI- and XPCI-CT-based tumour volumes (TV) for the MB350 group. The table reports the animal name, the survival counted from the day of glioblastoma implant, XPCI-CT and MRI tumour volumes normalized to the full brain volume with errors and the compatibility evaluation of MRI-XPCI-CT values together with the statistical indicators P(t) and the confidence level (C.L.). MRI-XPCI-CT tumour volumes with C.L.>5% are considered compatible each other. The last raw reports the average of the columns.

Sample name	Survival (days)	XPCI-CT T_V (%)	MRI T_V (%)	XPCI-CT – MRI compatibility		
				t	P(t) (%)	C.L. (%)
**B33**	15	0.84 ± 0.07	0.71 ± 0.11	1.04	61.6	38.4
**B34**	15	0.45 ± 0.03	0.42 ± 0.07	0.38	27.4	72.6
**B35**	15	0.41 ± 0.03	0.62 ± 0.10	2.01	94.0	6.0
**B36**	15	0.30 ± 0.02	0.58 ± 0.09	2.93	99.6	0.5
**B37**	15	0.26 ± 0.02	0.17 ± 0.03	2.36	96.2	3.8
**B23**	16	0.78 ± 0.07	0.76 ± 0.12	0.15	10.3	89.7
**B24**	16	0.88 ± 0.07	1.1 ± 0.2	1.26	73.3	26.7
**B27**	16	0.92 ± 0.08	1.2 ± 0.2	1.27	76.2	23.8
**Average**		0.61 ± 0.08	0.69 ± 0.13	1.43	80.6	19.4
